# Chemical Behavior of Mo_2_ *TM*B_2_ (*TM* = Fe, Co, Ni) upon the Oxygen Evolution Reaction (OER)

**DOI:** 10.1021/acsmaterialsau.5c00035

**Published:** 2025-06-24

**Authors:** Fatma Aras, Ulrich Burkhardt, Alim Ormeci, Horst Borrmann, Simone G. Altendorf, Yuri Grin, Iryna Antonyshyn

**Affiliations:** † Max-Planck-Institut für Chemische Physik fester Stoffe, Nöthnitzer Str. 40, Dresden 01187, Germany; ‡ Fritz-Haber-Institut der Max-Planck-Gesellschaft, Faradayweg 4-6, Berlin 14195, Germany

**Keywords:** intermetallic compound, boride, oxygen evolution reaction, electrochemical behavior, oxidation/reduction

## Abstract

The (electro)­chemical behavior of intermetallic compounds Mo_2_ *TM*B_2_ (*TM* = Fe, Co, Ni) under OER conditions has been investigated using electrochemical data combined with extensive bulk- and surface-sensitive material characterization. *In situ* formation of *TM*-rich amorphous layers, composed of oxides and hydroxides, accompanied by partial dissolution of molybdenum and boron, was observed for all three compounds. The degree of molybdenum and boron dissolution also influences the electronic state of *TM*s in their oxides/hydroxides formed on the surface of Mo_2_ *TM*B_2_. The *in situ*-formed Fe_2_O_3_ and Ni­(OH)_2_ on the surface of Mo_2_FeB_2_ and Mo_2_NiB_2_, respectively, are the origin of surface passivation and their OER inactivity. At the same time, the simultaneous presence of Co_3_O_4_ and Co­(OH)_2_ on the surface of an OER-exposed Mo_2_CoB_2_ electrode allows for the start of OER at a lower overpotential (ca. 290 mV) compared to elemental Co (ca. 370 mV), revealing better electrocatalytic activity. Extensive characterization of these materials as well as variation of the experimental conditions extends our understanding of the chemical properties of intermetallic compounds, which are of clear importance for their possible application as efficient electrocatalysts.

## Graphical abstract

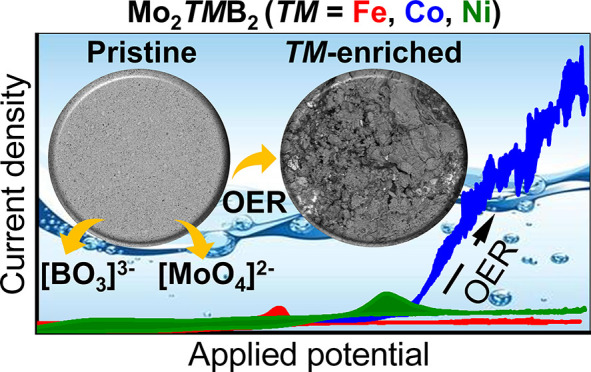

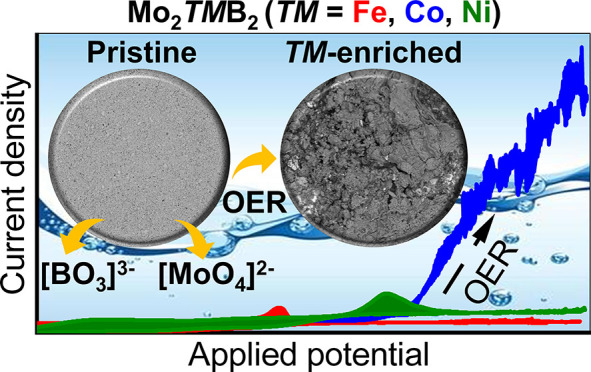

## Introduction

The development of sustainable energy technologies is vital to meet the escalating global energy consumption.^1,2^ Among sustainable energy solutions with net-zero carbon emissions, water electrolysis powered by renewable energy sources plays an essential role in the advancement of hydrogen-based energy conversion. Although hydrogen evolution is a well-established half-reaction of water splitting, the sluggish oxygen evolution (OER) is the bottleneck of this process.^3–5^

Over the past decades, there has been a great endeavor to discover/optimize/modify OER electrocatalysts, possessing improved efficiency and mechanical and chemical stability, as well as simple and inexpensive synthesis routes.^6,7^ Due to the low abundance and high price of noble metals, the industrial application of electrolyzers requires the development and/or optimization of OER electrocatalysts containing earth-abundant metals.^8–11^ In this respect, electrocatalysts with iron, nickel, and cobalt have great potential,^12,13^ and their oxides with high material stability in alkaline media have been extensively investigated.^6,14,15^ In addition, quantum chemical calculations clearly highlight cobalt and nickel (among the 3*d* transition metals) having optimal binding energies for intermediates in OER.^16,17^ This was also comprehensively confirmed by experimental studies.^15,18^

Under harsh OER conditions, the majority of electrocatalysts undergo structural and material transformations and should be considered as precatalysts (precursors).^19,20^ In the case of cobalt- or nickel-based anode materials, the *in situ*-formed cobalt or nickel (oxy)­hydroxides are thought to enable reversible electron transfer in the electrochemical double layer.^21,22^ In addition, the intentional and incidental Fe incorporation into Co- and Ni-based catalysts enhances their OER activity and stability.^5,22–26^ Based on such experimental and theoretical studies, Ni_1–*x*_Fe*_*x*_* OOH and CoOOH are regarded as the most active OER electrocatalysts in alkaline media.^20,27,28^ These findings paved the way for further investigation of Fe-, Co-, and Ni-based materials as alkaline OER electrocatalysts.

On one hand, tuning the *d*-orbital filling of transition metals (*TM*s) by modification of the coordination environment of electroactive species (e.g., through metal–metalloid/nonmetal interactions) is an actively studied strategy to improve the OER activity.^11^ In particular, *TM*-containing intermetallic compounds are considered promising precatalysts due to their, at least partially, ordered structures and unique bonding properties, which give rise to specific adsorption mechanisms of the reactant molecules as well as charge transfer during redox processes.^29–32^ On the other hand, the role of metalloids (C, B, S, P, As, Te) in OER electrocatalysts has been extensively studied in the last decades.^10,33^ The electron back-donation from metalloids to *TM*s is considered the most likely way to optimize the adsorption energies and consequently enhance OER activity.^34^ Particularly, numerous studies have been devoted to binary borides due to their thermodynamic stability, low cost, and sufficient electrical conductivity.

Ultrathin amorphous Ni*_*x*_* B nanosheets show different OER activities depending on the synthesis route (e.g., the overpotential to reach a current density of 10 mA cm^–2^ (η_10_) is equal to 380 mV for Ni*_*x*_* B annealed at 300 °C). Furthermore, improved OER activity was observed for Ni*_*x*_* B supported on the nickel foam (η_10_ = 280 mV).^35^ In comparison with Ni_2_P and Ni_3_S_2_, Ni_2_B exhibited the highest intrinsic OER performance, attributed to a thinner layer of the *in situ*-formed hydroxides (enabling faster electron transfer) and the promoting effect of Fe (from KOH electrolyte).^33^ With Co_3_B nanoparticles, an OER onset overpotential of 320 mV was achieved.^36^ Amorphous Co_2_B reveals an OER activity (η_10_ is equal to 380 mV), which can be maintained in 0.1 M KOH at a current density of 10 mA cm^–2^ for 60 h.^37^ Further improvements were made by its annealing at different temperatures and using various supports. Among crystalline Co*_n_* B (*n* = 1–3), Co_2_B requires the lowest overpotential of 287 mV to achieve a current density of 10 mA cm^–2^ in 1 M KOH. Furthermore, 12 h of chronopotentiometry at a current density of 50 mA cm^–2^ proved the robustness of Co_2_B.^38^

In addition to binary borides, ternary alloys and compounds with transition metals and boron came into consideration. Solid solution (Ni*_*x*_* Co_1–*x*_)_2_B (*x* = 0.25–0.75) was comprehensively investigated; (Ni_0.75_Co_0.25_)_2_B exhibited the best OER activity (η_10_ = 224 mV).^39^ Bifunctional Co–Ni–B nanoparticles reduced chemically on nickel foam possess an OER overpotential of 313 mV at a current density of 10 mA cm^–2^.^40^ Noticeable activity in both HER and OER was also evidenced for Co–W–B nanoparticles on nickel foam, which were prepared via an electroless plating method.^41^ Iron-containing cobalt borides facilitating higher catalytic activity than pure Co_2_B are also examples of improved OER performance.^42^ Ternary AlFe_2_B_2_ with a low OER overpotential (η_10_ ∼ 240 mV) maintained its electrocatalytic activity for at least 10 days. At increased current densities of 100 and 300 mA cm^–2^, the catalyst required only 290 and 320 mV, respectively. Partial leaching of Al from AlFe_2_B_2_ and the formation of Fe_3_O_4_ nanoclusters on the exposed [Fe_2_B_2_] layers were suggested as *in situ* modifications of this material, responsible for the outstanding OER activity, overcoming the activity of unsupported Fe_3_O_4_ and FeB.^43^

The ability of W, Mo, Nb, Ta, and Re to modify the electronic structure of neighboring atoms has led to an upward trend in studies of Fe-, Co-, and Ni-based materials with these metals. Among sol-gel-prepared NiFe*X* and FeCo*X* catalysts (*X* = Mo, W, Re, Ta, Nb, MoW), NiFeMo exhibited the highest OER activity and reasonable stability. Even under industrial conditions (30% KOH, 85 °C), the current densities of the NiFeMo electrode (at various applied cell potentials) exceeded those of the cell with the commercial Raney Ni electrode.^44^ In another study, a powder of ternary Mo_2_NiB_2_ was prepared by the sintering of constituent powders and coated on the glassy carbon electrode. Here, η_10_ is equal to 280 mV, which is lower than those of the corresponding binary borides (350 mV for Ni_2_B and 416 mV for MoB). Partial Mo leaching during electrochemical experiments leads to the *in situ* formation of OER-active nickel oxides/hydroxides with the remaining Mo oxides.^45^

In this study, the chemical behavior of the bulk ternary compounds Mo_2_ *TM*B_2_ (*TM* = Fe, Co, Ni) under the OER conditions in KOH electrolyte has been investigated using different electrochemical techniques, bulk- and surface-sensitive characterization, and computational insights into their electronic structure and chemical bonding. The drawbacks of drop-casted electrocatalysts and the use of binders have been overcome in this study by straightforwardly using the unsupported intermetallic compounds as electrode materials.

## Experimental Section

Intermetallic compounds Mo_2_ *TM*B_2_ (*TM* = Fe, Ni) were prepared from Mo slugs (Alfa Aesar, 99.95% metal basis), transition metal foils (Fe, Alfa Aesar, Puratronic, 99.995% metal basis; Ni, Chempur, 99.9+% metal basis), and B crystalline powder (Alfa Aesar, 99.99% metal basis). These elements were mixed in an atomic ratio of 2:1:2 (Mo:*TM*:B) and melted using an arc melter with a water-cooled copper mold under the partial pressure of argon. To ensure the sample homogeneity, the arc melting of ingots with turn-up was performed several times. The obtained ingots were placed in ZrO_2_ crucibles enclosed in tantalum tubes under argon to avoid any oxidation at elevated temperatures. Further homogenization was carried out by the annealing of as-cast Mo_2_FeB_2_ (1300 °C, 7 days) and Mo_2_NiB_2_ (1300 °C, initially 7 days, afterward extended to 21 days to achieve the equilibrium state and better crystallization) samples in high-temperature furnaces (HTM Reetz GmbH) located in an argon-filled glovebox (O_2_ and H_2_O content below 0.1 ppm).

In the case of Mo_2_CoB_2_, a different synthesis route was chosen. The direct arc melting of the components leads to significant amounts of Mo–B secondary phases. Therefore, to avoid the direct reaction of Mo with B, the Co–B precursor was synthesized and afterward reacted with Mo in the arc melter. For this purpose, Co and B powders (Co, Alfa Aesar, Puratronic, ∼22 mesh, 99.998% metal basis; B, Chempur, 99.99% metal basis) were weighed in the 1:2 ratio, mixed, and cold-pressed. The obtained pelletized specimen was placed in a ZrO_2_ crucible, sealed in a tantalum tube and quartz ampoule. Annealing of the Co–B precursor was executed at 1000 °C for 7 days, followed by quenching. The obtained Co–B precursor was arc-melted with Mo slugs (considering the target element ratio of 2:1:2), and the obtained ingot was annealed at 1400 °C for 7 days.

To manufacture unsupported electrodes, the annealed Mo_2_ *TM*B_2_ ingots were ground, and the obtained powders were sintered in a graphite die by spark plasma sintering (SPS, 515 ET Sinter Lab, Fuji Electronic Industrial Co. Ltd.). The sintering conditions were selected as follows: heating up to 1100 °C (*T*_max_), dwelling at *T*_max_ for 15 min, followed by cooling down to room temperature by turning off the furnace. The sintered samples were obtained in the form of a cylinder with a diameter of about 8 mm. The manufactured specimens were then polished using subsequently SiC paper and lubricant mixed with diamond colloidal solutions (with different particle sizes of 6, 3, and 1/4 μm) and a polishing machine (EcoMet250Pro, Buehler). The polishing of the electrode surface was repeated before each electrochemical experiment.

Phase analysis of the as-cast and annealed samples was carried out using powder X-ray diffraction (PXRD) data (Huber Imaging Plate Guinier Camera G670, Co *K*α_1_ radiation, λ = 1.78896 Å for Co- and Fe-containing samples or Cu *K*α_1_ radiation, λ = 1.54060 Å for Ni-containing samples). The experimental and calculated PXRD patterns were compared using WinXPow software.^46^ The lattice parameters of the identified phases were refined from the data set of the reflection positions (PXRD patterns with LaB_6_ (*a* = 4.15692(1) Å) as the internal standard) using the WinCSD software package.^47^ The electrodes after the electrochemical (EC) experiments were analyzed by X-ray diffraction in reflection mode (STOE Stadi MP diffractometer, DECTRIS MYTHEN 1K detector, Cu *K*α_1_ radiation, λ = 1.54060 Å). PXRD analysis of the as-cast samples revealed multiphase samples with the target phase Mo_2_ *TM*B_2_ and numerous secondary binary and ternary compounds (e.g., MoB, Mo_2_B, Co_2_B, Mo_3_ *TM*B_3_), revealing a nonequilibrium state of the ingots after arc melting. Further, homogenization heat treatment was therefore carried out at 1300–1400 °C (see above) based on thermal analysis, revealing absence of any thermal effects up to 1500 °C (see below). The detailed PXRD data for annealed samples are presented in the Results and Discussion.

Differential scanning calorimetry (DSC) under an argon atmosphere (Netzsch DSC 404C Pegasus, ZrO_2_ capped crucible, *T*_max_ = 1773 K, heating/cooling rate of 10 K min^–1^) was employed to determine the formation temperatures of Mo_2_ *TM*B_2_.

The morphology and homogeneity of the as-prepared samples were examined by bulk-sensitive techniques, including light microscopy (LM-Zeiss Axioplan 2 light microscope, CCD camera, and Olympus stream software^48^) and scanning electron microscopy (SEM, JEOL JSM-7800F microscope) with an energy-dispersive X-ray spectroscopy (EDXS) option (Quantax 400, X-Flash 6|30 silicon drift detector, Bruker). The compositions of the observed phases were determined via wavelength-dispersive X-ray spectroscopy (WDXS, CAMECA electron microprobe SX100 setup, W cathode, Mo 100% for Mo, FeSi for Fe, Ni_3_B for Ni and B, and Co 100% for Co were used as references). To track the morphological and compositional changes of Mo_2_ *TM*B_2_ after the electrochemical experiments, the backscattered electron (BSE), secondary electron (SE) images, and elemental mappings were recorded. The images were processed using Esprit 2.3 software.^49^

The electronic state of the constituent elements in the samples before and after the OER experiments was determined by X-ray photoemission spectroscopy (XPS). The spectrometer is equipped with a twin crystal monochromatized Al Kα source (*hv* = 1486.6 eV) and a Scienta R3000 electron energy analyzer in normal emission geometry. All measurements were performed at room temperature, and the pressure in the spectrometer chamber was maintained in the 10^–10^ mbar range. The overall energy resolution was approximately 0.4 eV, and the Fermi level was calibrated with respect to a polycrystalline Ag reference.

Electrochemical experiments were performed in 1 M KOH electrolyte (Thermo Fisher Scientific) at room temperature using the PEEK three-electrode house-made cell (Figure S1a) with a Mo_2_ *TM*B_2_ working electrode, a Hg/HgO reference electrode (PINE Research, 9.5 mm replaceable threaded frit tip, stored in 1 M KOH), and a Pt wire counter electrode (PINE Research, coil with 4.9 cm^2^ surface area). The Hg/HgO reference electrode was calibrated against the reference hydrogen electrode (RHE, Gaskatel, HydroFlex) prior to each electrochemical measurement. The electrolyte solution was deaerated with Argon 5.0 for at least 20 min before each electrochemical experiment. SP-300 and VMP-300 potentiostats (Bio-Logic Science Instruments) were used for EC measurements and data recording. Data analysis was performed using EC-Lab^50^ and Origin^51^ software.

The electrochemical measurements were based on the following techniques: (i) open-circuit voltage (OCV) to release the system, (ii) linear sweep voltammetry (LSV) to support a smooth transition from the OCV potential to the cyclic voltammetry (CV) starting potential, (iii) CV either for surface pretreatment and monitoring of the electrochemical properties at potentials below the OER or for OER activity assessment (above 1.2 V versus RHE), and (iv) chronopotentiometry (CP) to follow the stability of the OER activity over time. The detailed description of the electrochemical workflow and measurement parameters is given in the Supporting Information (The optimized atomic coordinates Figure S1b). The Fe concentration in the used KOH electrolyte is below the detection limit of inductively coupled plasma-optical emission spectrometry (ICP-OES, <0.01 mg L^–1^). Current densities were obtained by dividing the measured current by the geometric area of the Mo_2_ *TM*B_2_ electrode (0.204 cm^2^). For all electrochemical measurements, 85% *iR* compensation was applied by the EC-Lab.

The concentration of dissolved components in the aliquots of the electrolyte taken after each EC was analyzed with ICP-OES (spectrometer Agilent 5100 SVDV).

The first-principles electronic structure calculations were performed by using the all-electron full-potential local orbital method (FPLO).^52^ Local (spin) density approximation (LDA/LSDA) as parametrized by Perdew and Wang were employed to account for the exchange–correlation effects.^53^ The atomic positions were optimized by fixing the lattice parameters to their experimentally determined values. The force criterion was adopted at 1 meV Å^–1^. The optimized atomic coordinates are given in Table S1. Brillouin zones were sampled by **k**-meshes of 18 × 18 × 18 and 12 × 12 × 22 for Mo_2_CoB_2_ (Mo_2_NiB_2_) and Mo_2_FeB_2_, respectively. Comparison with the valence band spectra obtained by XPS measurements was achieved through the projected densities of states (pDOS) computed for the occupied states in the respective free atoms. The free-atom valence electron configurations used are as follows: Mo (5*s* ^1^, 4*d* ^5^), Ni (4*s* ^2^, 3*d* ^8^), Co (4*s* ^2^, 3*d* ^7^), Fe (4s^2^, 3*d* ^6^), and B (2*s* ^2^, 2*p* ^1^). The pDOS of these states were first broadened by a Gaussian function of width 0.50 eV and processed with the Fermi–Dirac distribution function with *T* = 300 K, and then their sum weighted by the corresponding photoionization cross sections^54^ was evaluated.

Using the optimized atomic coordinates and experimentally obtained lattice parameters, the electron density (ED) and the electron localizability indicator (ELI-D) were calculated with a specialized module implemented in the FPLO program package.^55^ The topology of ED and ELI-D was analyzed with the DGrid program.^56^ The ED was integrated within atomic and bond basins, i.e., spatial regions confined by zero-flux surfaces in the gradient field of ED and ELI-D, respectively. This technique represents the procedure proposed in the Quantum Theory of Atoms in Molecules (QTAIM^57^) and provides effective electron populations for the QTAIM atoms and ELI-D bond basins. Further information about the bonding between atoms is obtained from the combined analysis of ED and ELI-D (electron localizability approach^58^).

## Results and Discussion

Mo_2_CoB_2_ and Mo_2_NiB_2_ are compounds isostructural to W_2_CoB_2_ (space group *Immm*, *a* = 7.087(2) Å, *b* = 4.564(2) Å, *c* = 3.164(5) Å for Mo_2_CoB_2_ and *a* = 7.075(5) Å, *b* = 4.557(5) Å, *c* = 3.179(5) Å for Mo_2_NiB_2_ ^59^), while Mo_2_FeB_2_ represents own structure type, “coloring” superstructure of the U_3_Si_2_ type (space group *P*4/*mbm*, *a* = 5.807(4) Å, *c* = 3.142(3) Å^60^). Comparison of the experimental PXRD patterns with the calculated ones reveals the single-phase nature of the Mo_2_FeB_2_ sample and the presence of secondary phases Mo_3_ *TM*B_3_ and impurity Mo*TM*Si in the case of the Mo_2_NiB_2_ and Mo_2_CoB_2_ samples (Figure 1). The refined lattice parameters (standardized according to ref 61) are as follows: *a* = 3.1658(2) Å, *b* = 4.5679(3) Å, *c* = 7.0907(4) Å (Mo_2_CoB_2_), *a* = 3.1846(1) Å, *b* = 4.5603(2) Å, *c* = 7.0872(2) Å (Mo_2_NiB_2_), and *a* = 5.7905(2) Å, *c* = 3.1462(2) Å (Mo_2_FeB_2_). They are very close to those published in the literature, cf. above.

**Figure 1:**
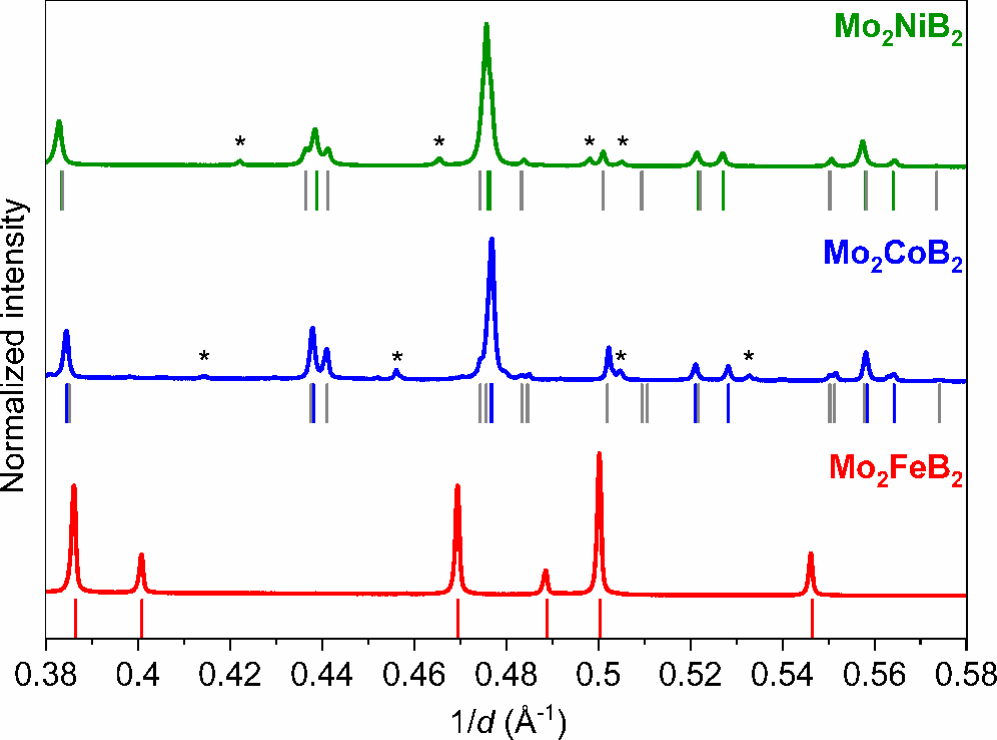
PXRD patterns of as-synthesized Mo_2_ *TM*B_2_ (*TM* = Fe, Co, Ni) samples (positions of reflections in the calculated PXRD patterns are presented with colored (for Mo_2_ *TM*B_2_) and gray (for Mo_3_ *TM*B_3_) ticks; peaks corresponding to traces of MoCoSi and MoNiSi impurities (MgZn_2_-type of structure) are marked by asterisks).

BSE images demonstrate the single-phase matrix of Mo_2_FeB_2_ with tiny traces of the (Fe_1–*x*_Mo*_*x*_*)_2_B admixture (undetectable by PXRD), which is formed due to small boron losses. In the case of Mo_2_ *TM*B_2_ (*TM* = Co, Ni), the target phases are in equilibrium with the Mo_3_ *TM*B_3_ phases in as-synthesized samples (Figure S2). According to the available isothermal section of the Mo–Co–B phase diagram at 1100 °C^62^ and the Mo–Ni–B one at 950 °C,^63^ Mo_2_CoB_2_ and Mo_2_NiB_2_ are in equilibrium with the ternary phases Mo_3_ *TM*B_3_ (first mentioned as Mo_3_ *TM*B_6_,^62^ but later considered as Mo_3_ *TM*B_3_ ^64^), and even small deviations from the nominal composition during the synthesis lead to the appearance of Mo_3_ *TM*B_3_. On the other hand, there is no information about the Mo_3_FeB_3_ phase in the Mo–Fe–B system.^62^

Furthermore, noticeable homogeneity ranges for Mo_2_ *TM*B_2_ were mentioned in the literature. The composition of Mo_2_FeB_2_ varies from 32 to 40 at. % Mo, 28 to 20 at. % Fe, and 40 at. % B,^60^ implying significant substitution of molybdenum by iron in the structure. Data on the homogeneity range for Mo_2_CoB_2_ are conflicting in the literature, indicating either a constant composition for this compound^65^ or a noticeable width of the homogeneity range in terms of metal content.^66^ For Mo_2_NiB_2_, a distinct homogeneity range in terms of Ni content in the total amount of metals (Ni/(Ni+Mo)) from 0.330 (Mo_40_Ni_20_B_40_) up to 0.356 (Mo_38.6_Ni_21.4_B_40_) has been reported.^66^ The compositions of the target phases and secondary phases in our study were determined by wavelength-dispersive X-ray spectroscopy (WDXS, Table 1). The obtained Fe content is within the compositional range reported in the literature,^60^ but the molybdenum-to-boron ratio is slightly different. Similarly, the Co contents of both phases (target Mo_2_CoB_2_ and secondary Mo_3_CoB_3_) agree well with nominal ones; however, the Mo/B ratio differs significantly from 1:1 (as expected from the nominal compositions). Contrary to the Co-containing phases, the Ni-containing phases show increased Mo/B ratios as well as slightly increased Ni contents (compared to the nominal compositions). Such deviations can be related to particularly challenging boron quantification by WDXS due to the non-negligible superposition of the low-energy Mo X-ray fluorescence lines *E*(Mo M*z*_1_) = 192 eV with the most intense B line *E*(B Kα_1_) = 183 eV^67^ and required suitable measurement routines to deconvolute the intensity contributions.

**Table 1:** WDXS Analysis of As-Synthesized Mo_2_ *TM*B_2_ (*TM* = Fe, Co, and Ni) Samples

	**target phase Mo**_**2**_ *TM* **B**_**2**_ **(at. %)**			**minority phase Mo**_**3**_ *TM* **B**_**3**_ **(at. %)**		
**samples**	**Mo**	***TM***	**B**	**Mo**	***TM***	**B**
**Mo**_**2**_ **FeB**_**2**_	44.3 (3)	21.1 (1)	34.6 (4)	none		
**Mo**_**2**_ **CoB**_**2**_	37.3 (1)	20.1 (1)	42.6 (2)	39.4 (4)	15.1 (7)	45.5 (7)
**Mo**_**2**_ **NiB**_**2**_	45 (2)	23 (1)	32 (3)	49 (2)	16 (1)	35 (3)

The crystal structures of Mo_2_ *TM*B_2_ compounds had been elucidated in the past, but there is no available information about the chemical bonding in these compounds. Therefore, detailed bonding analysis was carried out to gain insights into interactions between atoms in these compounds and their possible impact on the chemical behavior of Mo_2_ *TM*B_2_ under OER conditions. The crystal structures of Mo_2_FeB_2_ and W_2_CoB_2_ types are traditionally interpreted by means of the inner part of the coordination polyhedron of boron.

According to this description, the metal atoms form triangular prisms [BMo_6_] or [BMo_4_ *TM*_2_] around each boron atom. The double prisms are stacked over triangular faces to columns, which, in turn, share side edges (Figure 2, top; cf. overview in ref 68). A more recent study on Ga_3_Fe, whose crystal structure is also built of such double prisms [FeGa_6_], showed that from chemical bonding point of view, a larger structural segment should be considered as the environment of the Fe-pair instead of individual Fe atoms—the rhombic prism that also includes additional atoms centering the side faces [(Fe_2_)­Ga_8_Ga_4_]. Atomic interactions in this structural segment define the band structure of the whole material.^69^ Applying this approach to the Mo_2_ *TM*B_2_ compounds would yield a different picture of the structures (Figure 2, middle). The questions, which of the two representations is supported from the point of view of atomic interactions and how the bonding picture helps to interpret experimental results, were the starting point for the investigation of chemical bonding in the three materials being the focus of the present paper. The electron localizability approach, a quantum chemical technique in position space,^58^ was employed.

**Figure 2:**
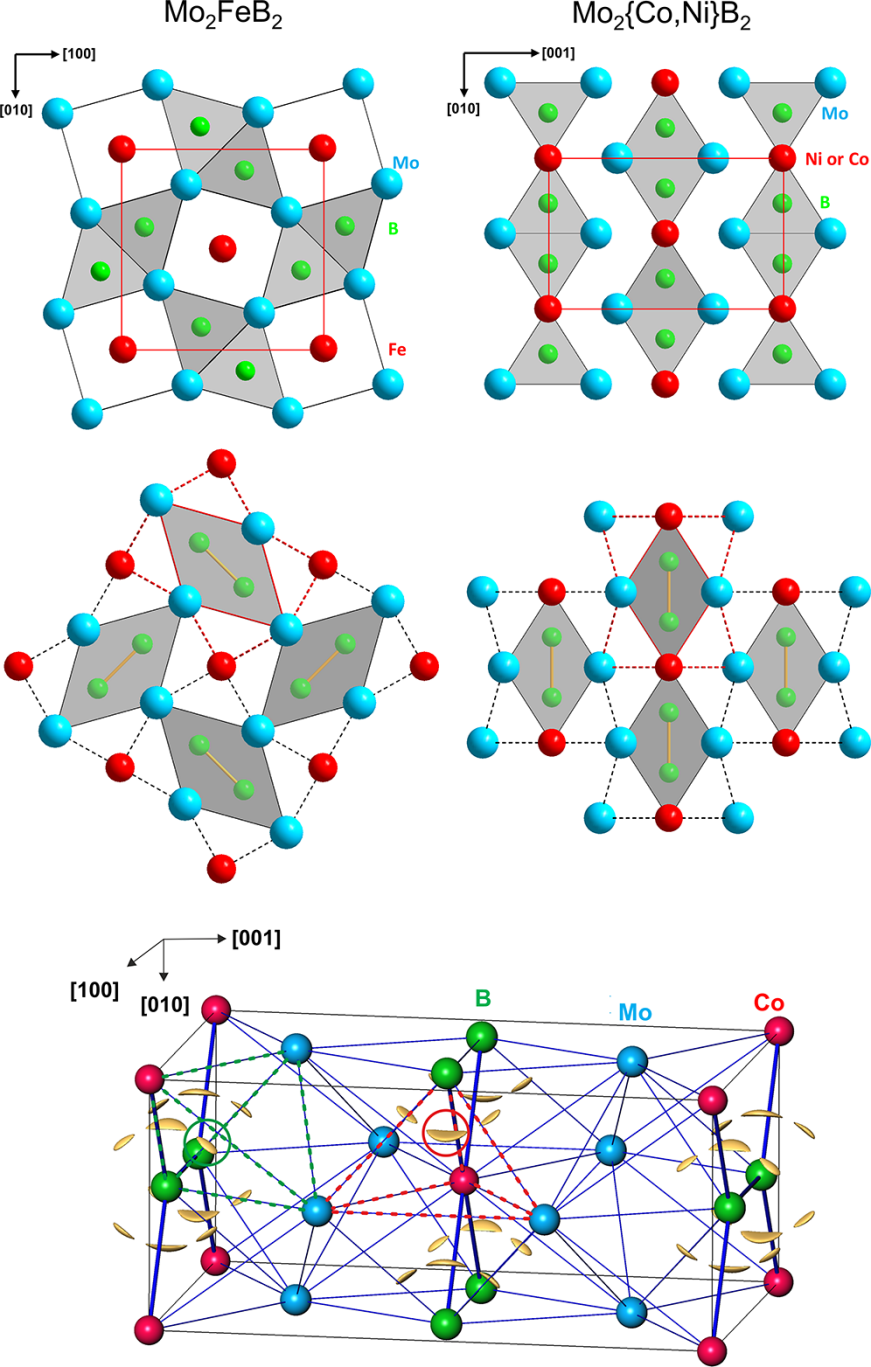
Crystal structure and bonding in Mo_2_ *TM*B_2_ compounds: (top) crystal structures of Mo_2_FeB_2_ (left) and Mo_2_{Co,Ni}­B_2_ (right) as packings of condensed trigonal prisms [B*M*_6_] formed by metals around boron atoms. (middle) Packings of side-centered rhombic prisms [(B_2_)*M*_8_ *M*_4_] around boron pairs in Mo_2_FeB_2_ (left) and Mo_2_{Co,Ni}­B_2_ (right) as obtained from the analysis of chemical bonding. (bottom) Isosurface of the electron localizability indicator with ELI-D = 1.28 visualizing the position of the 4*a*-B–Co–Mo–Mo bonds in the Mo_2_CoB_2_ structure and illustrating largely homogeneous character of bonding in these materials.

The effective charges evaluated from the electron density within the QTAIM approach^57^ reveal only a moderate charge transfer in all three materials, if comparing with OER active ternary compound Hf_2_B_2_Ir_5_.^19^ Molybdenum carries the most positive charge in all three compounds, which is practically independent of the *TM* as the third component and the type of the crystal structure (+0.75, +0.79, and +0.77, for Fe, Co, and Ni compounds, respectively). Quite the same is valid for the negative effective charges of boron (−0.79, −0.65, and −0.64, respectively), where the small charge difference can be considered for the compounds with different structure types (Mo_2_FeB_2_ versus isotypic Mo_2_CoB_2_ and Mo_2_NiB_2_, respectively). The most essential difference is evaluated for the *TM* atoms. While in W_2_CoB_2_-type compounds, they show small negative charges (−0.30 for Ni and −0.28 for Co), in Mo_2_FeB_2_, iron already shows a minor positive charge of +0.08. Thus, also here, the overall atomic arrangement influences the charge transfer rather than the position of *TM* in the Periodic Table. Further information about the chemical bonding was obtained from the analysis of the topology of the electron localizability indicator (ELI-D). While applying such an approach to electron density yields shapes and charges of atomic species, in the case of ELI-D, this technique yields the space portioning into regions, representing bonds, lone pairs, or inner shells of the atoms with the corresponding electronic populations.^58^ Characteristic for all three investigated compounds, the two-atomic bonds are very weakly pronounced (the populations of Mo–*TM* or Mo–Mo bond basins are below 0.10 e^–^), and there are no dedicated basins for Mo–B interactions (Figure S3). The B–B basins represent de facto six-atomic contributions. Besides two boron atoms, four molybdenum species contribute to them (approximately 20%). Therefore, the population of this bond is small for Ni and Co compounds (1.69 and 1.62 e^–^, respectively) and increases slightly to 1.89 e^–^ for Mo_2_FeB_2_. All other bonds in these three compounds are four-atomic. Boron is the main contributor to all 4*a*–B–*TM*–Mo–Mo bonding basins with large populations. The four-atom basins formed only by Mo and *TM* show essentially lower populations (Figure S3).

The 4*a*-bonds play an important role in rhombic prisms [(B_2_)*M*_8_ *M*_4_]. The volume of these structural segments is nearly completely formed by the 4*a*-basins of the B–*TM*–Mo–Mo bonds (red and gray for Mo_2_CoB_2_ and red and light pink for Mo_2_FeB_2_ in Figure S4). The 4*a* metals-only bonds are located on the periphery of the rhombic prisms (violet for Mo_2_CoB_2_ in Figure S4). The 2*a*-basins of the *TM*–Mo bonds form the contact points between the neighboring rhombic prisms (orange, Figure S4). Thus, from the bonding point of view, the crystal structures of the investigated compounds should be represented as packings of the side-centered rhombic prisms (Figure 2, middle) rather than as traditionally used packings of triangular prisms.

Further analysis of the ELI-D distribution using the isosurfaces of ELI-D with the values close to maximum (which visualize the position of the ELI-D maxima) reveals that the unit cell volume of all three compounds can be understood as formed by the volume of the 4*a*-bonds (cf. Mo_2_CoB_2_ in Figure 2, bottom). Therefore, the bonding in the compounds studied does not show striking features in distinct directions and should be considered homogeneous. As a consequence, no special cleaving directions by materials grinding are expected. The so-obtained surfaces should have rather similar atomic decoration and not differ strongly in their chemical behavior.

Having a comprehensive insight into the bulk features of pristine Mo_2_ *TM*B_2_ compounds from experimental and theoretical points of view, it is also important to add information about the surface state before their investigation as OER electrode materials. For this purpose, laboratory X-ray photoelectron spectroscopy (XPS) was implemented. The binding energy of the Fe 2*p*_3/2_ core level (707.1 eV) of Mo_2_FeB_2_ (Figure 3a) reveals that there is no significant chemical shift compared to the published data for elemental Fe (706.9 eV^70^, 707.0 eV,^71^ 707.1 eV^72^) and the binary compounds FeB and Fe_2_B (707.2 and 707.1 eV, respectively^70^). Furthermore, the negligible shift of the Fe 2*p*_3/2_ core level is in line with the negligibly small QTAIM positive charge of Fe (+0.08) in Mo_2_FeB_2_. In addition, iron oxides were detected on the surface due to partial surface oxidation upon exposure to air. The doublet of broad, asymmetric Fe 2*p* peaks for oxides is centered around 710.3 eV (2*p*_3/2_) and 724.4 eV (2*p*_1/2_), together with the corresponding satellite peaks (Figure 3a). The peak positions align with the published values of Fe 2*p*_3/2_ (710.4 eV) in Fe_2_O_3_.^71,73^ Different iron oxides have subtle differences in Fe 2*p* core levels,^73^ but, in addition to the Fe 2*p* core levels, valence band features as well as O 1s core level XP spectra were compared to determine the nature of the oxidic species. Fe_2_O_3_ is dominant among contributing iron oxides on the pristine Mo_2_FeB_2_ surface (Figures 3a and S5a). In Mo_2_CoB_2_, the shift of the Co 2*p*_3/2_ core level toward higher binding energy (778.6 eV) was evident from the comparison with literature values for elemental Co (778.0 eV^74^ and 778.4 eV^72^). In terms of partial surface oxidation, the absence of cobalt oxide signals in the XP spectra reveals higher resistance of Mo_2_CoB_2_ to oxidation compared to Mo_2_FeB_2_ (Figures 3a and S5b). Finally, the maxima of the Ni 2*p*_3/2_ core level in Mo_2_NiB_2_ is centered at 853.6 eV (Figure 3a), which is almost 1 eV higher in binding energy than the published data for elemental Ni (852.5 eV^74^ or 852.8 eV^72^). Mo_2_NiB_2_, also, showed essentially no surface oxidation under air (Figures 3a and S5c). Although the shift toward higher binding energies of Co 2*p* and Ni 2*p* (compared to elements) suggests positively charged *TM* atoms, the QTAIM analysis has revealed negative charges on Co and Ni atoms. A similar situation was encountered in the HAXPES study of Al–Pt binary compounds.^75^ Pt atoms in Al–Pt binary compounds are negatively charged, but the Pt 4*f* core levels shift to higher binding energies. The explanation was based on the observation that the number of Pt 5*d* electrons in the binaries, computed by using the ELI-D analysis, was found to be less than the corresponding value computed for elemental fcc Pt. It was argued that 5*d* electrons are more effective in the screening of the core hole, and if their numbers decrease, then screening will be weaker and the core levels will shift to higher binding energies. The same approach applied to Co and Ni yields a similar scenario: 3*d* occupancies in hcp Co and fcc Ni are 6.66 and 7.80 electrons, respectively, whereas they are 6.59 and 7.62 electrons in Mo_2_CoB_2_ and Mo_2_NiB_2_, respectively. Therefore, there are fewer 3*d* electrons in the ternaries, providing weaker screening; this results in higher binding energies. The XP Mo 3*d* core level spectra with the main contributions at binding energies of 228.1 and 231.3 eV, corresponding to the Mo 3*d*_5/2_ and Mo 3*d*_3/2_ doublets, respectively, indicate the presence of intermetallic molybdenum (Mo^δ+^), with a positive shift of 0.3 eV (estimated for 3*d*_5/2_) with respect to elemental Mo in the literature (227.8 eV^76^). Only in the case of Mo_2_FeB_2_, there is an additional contribution from MoO_3_ (Figures 3b and S5a), which has also been recognized in other studies with molybdenum-based materials.^77^ XP spectra of the B 1*s* core levels reveal B^δ−^ in Mo_2_ *TM*B_2_ (188.3 eV), possessing ca. 0.2 eV shift to the lower binding energy compared to the B 1*s* core level in metallic boron (188.5 eV^78^). Exclusively on the Mo_2_FeB_2_ surface, boron oxide (peak at 192.9 eV) is also present (Figures 3c and S5a). Very clear evidence of the positively charged Mo and negatively charged B in all Mo_2_ *TM*B_2_ compounds agrees with the corresponding QTAIM effective charges presented above.

**Figure 3:**
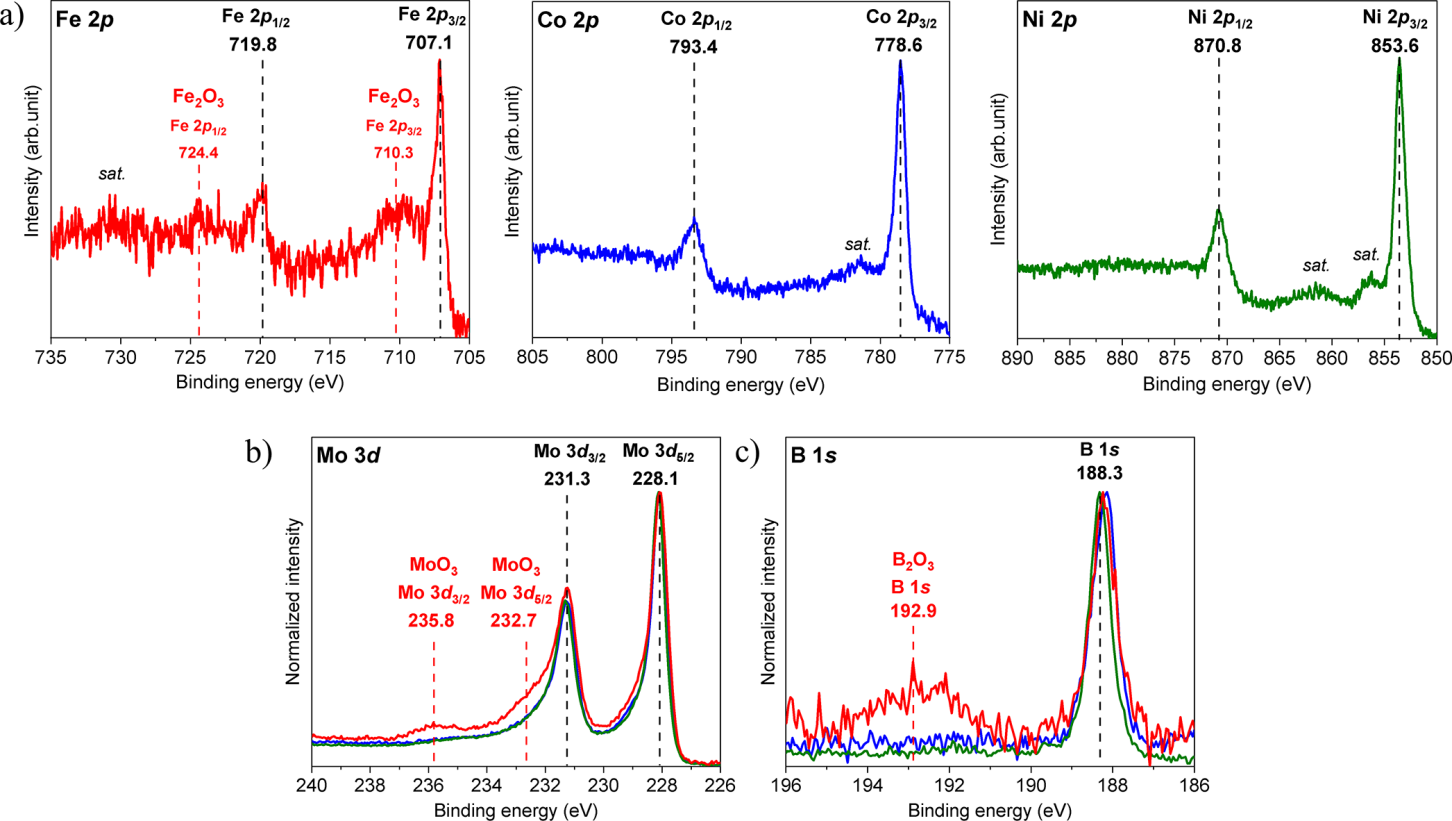
XP spectra of *TM* 2*p* (a), Mo 3*d* (b), and B 1*s* (c) core levels in pristine Mo_2_ *TM*B_2_ (*TM* = Fe (red), Co (blue), and Ni (green)). Dashed vertical lines as well as numerical values represent the binding energies at the maximum intensity for major XP peaks. The peaks of surface oxides are labeled in red. XP spectra of Mo 3*d* (b) and B 1*s* (c) are normalized to 1 for a better comparison of their features.

The experimentally measured valence band spectra for pristine Mo_2_ *TM*B_2_ clearly reveal similarities (Figure S6a). Furthermore, these XPS data are compared with the computed valence band spectra in Figure S6b–d (for details, see the Experimental Section). The underlying DOS and pDOS are presented in Figure S7. The XP valence band spectra within ∼4 eV of the Fermi energy are reproduced rather well. The peaks around 11 eV binding energy in the experimental spectra for Mo_2_CoB_2_ (Mo_2_NiB_2_) appear around 9 eV in the LDA calculation, and they are largely due to B 2*s* states (Figure S7). Still, the calculation captures the feature that the peak is relatively more pronounced in Mo_2_CoB_2_ than that in Mo_2_NiB_2_. Since the Mo_2_FeB_2_ data look quite noisy for binding energies larger than 10 eV, no such peak is discernible in the Mo_2_FeB_2_ spectra. However, the LSDA calculation finds one shoulder at ∼6 eV, and two peaks centered around 7.5 and 10 eV (Figure S6b). The shoulder is mainly due to the Mo 4*d* states hybridizing with the B 2*p* states, whose photoionization cross section is 2 orders of magnitude smaller. The two peaks are again due to B 2*s* states, and the reason for the split is the presence of a short B–B contact in Mo_2_FeB_2_. This short contact (∼1.79 Å) gives rise to clearly separated bonding and antibonding bands.

The chemical behavior of Mo_2_ *TM*B_2_ under oxygen evolution reaction conditions was investigated at ambient conditions in 1 M KOH. Prior to OER activity estimation, the electrochemical pretreatment of the samples was executed by cyclic voltammetry in two ways: (i) at the anodic potentials close to OER with high scan rates (protocol 1) or (ii) in the potential range between HER and OER with reduced scan rates (protocol 2, adopted from refs 8,14,79 ; Figure S1b).

CVs with Mo_2_ *TM*B_2_ electrodes at anodic potentials close to the OER show a pronounced current density that varies with the increasing number of cycles, *N*_c_ (Figure S8). A broad oxidation peak at around 1.17 V versus RHE on the first CV with the Mo_2_FeB_2_ electrode corresponds to the Mo^3+^/Mo^4+^ transition^80,81^ and reveals the pronounced anodic oxidation and accompanying dissolution of molybdenum (Figure S8a). As *N*_c_ increases, the oxidation peak becomes broader and less intense, and its position is shifted toward higher potentials, i.e., the surface oxidation becomes slower and more energy-demanding due to the increased thickness of the *in situ*-formed oxide layer. After 300 CV cycles, significantly decreased and almost constant values of the current density were achieved above 1.35 V versus RHE, revealing significant changes during the sample pretreatment and, finally, a partially equilibrated state of the electrode. Also, the absence of a reduction peak points out that the surface has been modified irreversibly. As a reference, the electrochemical behavior of elemental Mo was investigated by applying the same reaction conditions. The oxidation peaks of Mo and Mo_2_FeB_2_ are observed in a similar potential range of CVs (Figures S8a and S9a) and correspond to Mo partial oxidation.^80,82^ The surface of the Mo electrode turned dark brown-black immediately after the first CV cycle, revealing a pronounced oxidation of molybdenum (with a broad oxidation peak shifting from 1.35 to 1.25 V with increasing *N*_c_), represented by significant current densities of hundreds of mA cm^–2^.^80,82^ The oxidation of Mo is also irreversible, as evidenced by the absence of a reduction peak in the cyclic voltammograms (Figure S9a). Contrary to Mo, other transition metals did not show such pronounced and irreversible oxidation features (Figure S9b–d). Due to the amorphous nature of the *in situ*-formed oxides/hydroxides, their characterization by XRD is hindered.

The CVs of Mo_2_CoB_2_ depicted characteristics similar to those of Mo_2_FeB_2_. With increasing *N*_c_, the potential of the Mo^3+^/Mo^4+^ anodic peak shifted to higher values, and the obtained current density increased up to 110 mA cm^–2^ (Figure S8b). However, the broad reduction peak at 1.37 V was observed, substantially enlarged, and shifted to the lower potential values upon cycling. The partial reduction in the case of Mo_2_CoB_2_ is the main difference from the irreversible changes on the Mo_2_FeB_2_ surface. The current density, obtained during CVs for Mo_2_NiB_2_, is significantly lower than that for the Fe- and Co-containing compounds, but still large enough to hinder the monitoring of the oxidation–reduction peaks of Ni in Mo_2_NiB_2_ (Figure S8c). Summarizing, the main contribution to the current densities in the oxidation region of all Mo_2_ *TM*B_2_ compounds is from partial irreversible Mo oxidation on the surface and its dissolution.

Since the ongoing irreversible oxidation determines the dissolution rates of the constituent elements and results in transport limitations depending on the thickness of the surface amorphous layer, the applied reaction conditions have a significant influence on the final state of the electrode surface. In order to reach the equilibrium in a shorter time, CV pretreatment was performed at lower potentials within a wider potential range at a slower scan rate (protocol 2, Figure S1b).

Especially, for Mo_2_FeB_2_ and Mo_2_NiB_2_, due to the very fast CV potential scanning (100 mV s^–1^, protocol 1), only a quasi-equilibrium state was reached after 300 CV cycles, whereas 50 CV cycles under the conditions of protocol 2 were sufficient to reach the steady state (unchanged CVs from almost the last 35th CV cycle, Figure 4a). Anodic dissolution of molybdenum in alkaline media at low potentials (0.6–0.9 V versus RHE) originates from the transition from Mo^0^ to Mo^3+^ species (Figure S10).^80^ Similar features are obvious in the CVs of Mo_2_CoB_2_ and Mo_2_NiB_2_, the Mo^δ+^/Mo^3+^ oxidation, with the appearance of broad peaks at 0.6–0.9 V versus RHE (Figure 4a). In the case of Mo_2_FeB_2_, the Fe^2+^/Fe^3+^ transition is found in the close vicinity of the Mo^δ+^/Mo^3+^ region, hampering the clear assignment of the oxidation currents to a certain electrochemical process. Nevertheless, the obtained high current density reflects the characteristic of Mo dissolution as observed in other intermetallic compounds. Furthermore, the broad peak at a low applied potential (0.3–0.6 V versus RHE) is thought to be due to the partially oxidized surface of the initial material. That is, further oxidation of the constituent elements may occur in a slightly lower potential range with respect to Co- and Ni-based materials.

**Figure 4:**
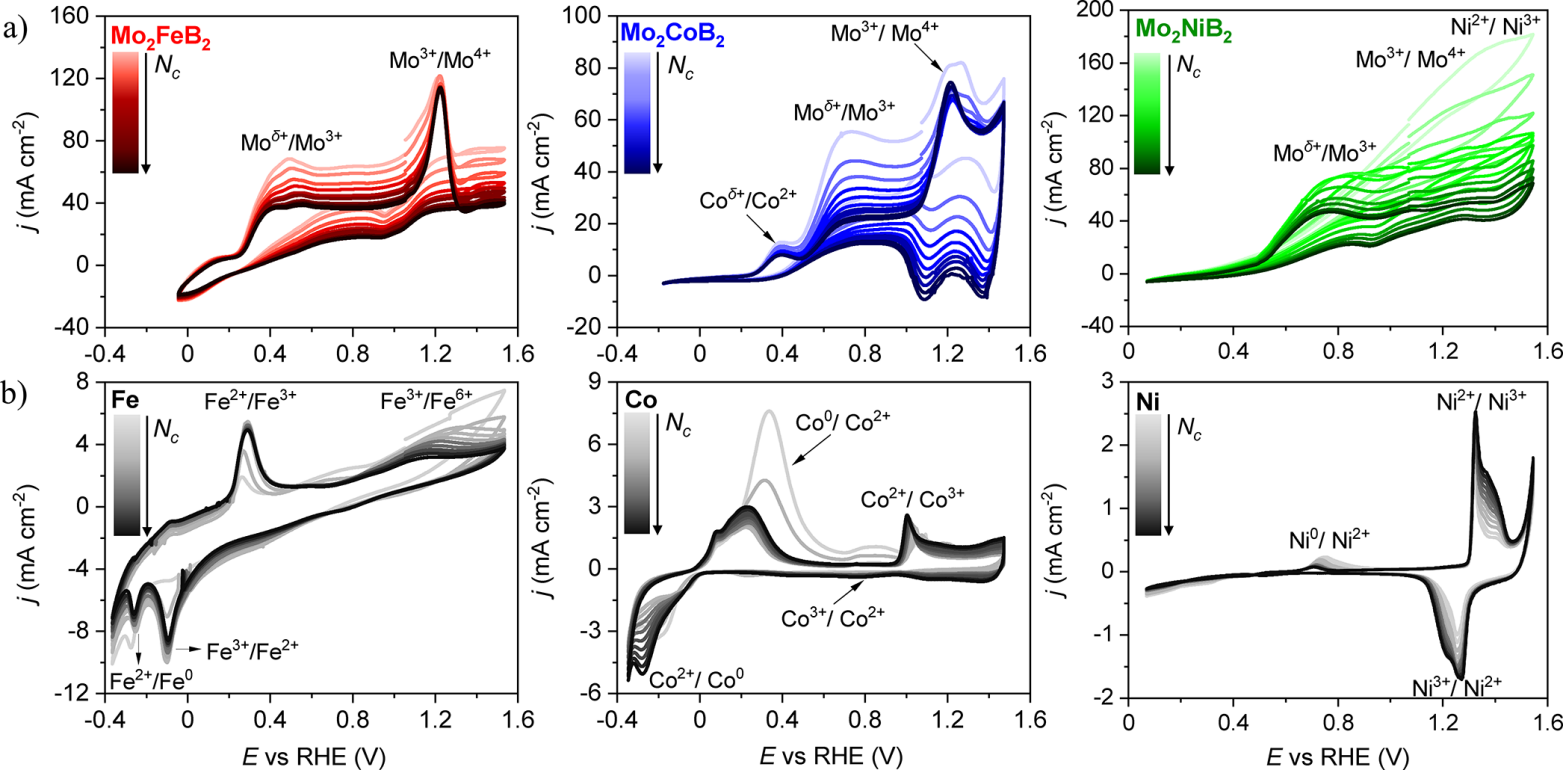
Cyclic voltammograms of Mo_2_FeB_2_ (red), Mo_2_CoB_2_ (blue), and Mo_2_NiB_2_ (green) (a) compared to those of reference Fe, Co, and Ni (b), measured with protocol 2. The increasing intensity of the CV curve colors corresponds to the increasing number of measured cycles (*N*_c_, every 5th cycle is presented, and overall *N*_c_ is 50). The oxidation/reduction peaks are labeled by assumed electrochemical processes based on the current study and literature overview.

With increasing *N*_c_, the current density of the Mo^δ+^/Mo^3+^ oxidation peak reaches a saturation, i.e., steady state. The second peak (at ca. 1.2 V versus RHE) is attributed to the Mo^3+^/Mo^4+^ transition, which was already observed in previous electrochemical experiments with elemental Mo and Mo_2_ *TM*B_2_ (Figures 4, S9a, and S10). The formation of transition metal oxides/hydroxides on the surface of elemental Fe, Co, and Ni is obvious from the corresponding CVs (Figure 4b); it is reversible in nature and is in good agreement with the experimental findings in the literature on other materials.^8,14,79,83,84^ Compared to elemental Fe, the Mo_2_FeB_2_ surface was severely affected by the formation of iron­(III) oxide and molybdenum oxidation at 1.2 V versus RHE, which probably leads to the formation of an oxide layer with limited electrical conductivity rather than OER-active species.^14^ A comparison of the CVs for elemental cobalt (reversible oxidation, Figure 4b) and Mo_2_CoB_2_ (irreversible surface change, Figure 4a) clearly shows the different characteristics of the surface oxidation (passivation).^79^ OER-active species with the Co^3+^ oxidation state (described in the literature as cobalt oxyhydroxide, CoOOH)^17,79,82,83^ are not clearly detected in CV cycles of Mo_2_CoB_2_ due to high current density corresponding to the oxidation of molybdenum ions. The observed reduction peaks of Mo_2_CoB_2_ (Figure 4a) are considered to belong to the reduction of high oxidation states of the Co species in the back scan. Reduction of solvated molybdenum ions is not considered because of the insignificant amount of molybdenum species on the surface (based on XPS and EDXS) as well as the irreversibility of Mo oxidation observed for elemental Mo (Figures S9a and S10). In the case of elemental nickel, an oxidation peak (∼0.75 V versus RHE) is attributed to the formation of a nickel oxide/hydroxide film of limited thickness,^85^ whereas the Ni^2+^/Ni^3+^ reversible redox transition takes place at 1.3–1.5 V. Measuring with the Mo_2_NiB_2_ electrode, the largest contribution to the anodic currents is from the Mo^δ+^/Mo^3+^ transition, whereas the Mo^3+^/Mo^4+^ and Ni^2+^/Ni^3+^ electrochemical processes are barely distinguishable in the last CV cycles (Figure 4a).

Due to the noticeable current densities even in the potential range of the material pretreatment, the offset current density resulting from the partial dissolution of constituent components is also expected in the OER region. Indeed, in the Mo_2_FeB_2_ case (pretreated according to protocol 1), the active anodic dissolution of molybdenum and boron leads to the enlarged current density values up to 65 mA cm^–2^ (Figure 5). The noticeable oxidation peak around 1.16 V corresponds to Mo oxidation and agrees with the data from the CV pretreatment (Figure 4a). Contrary to elemental Fe (Figure S11b), no hint of OER activity with the Mo_2_FeB_2_ electrode was observed in the potential range of 1.23 to 2.0 V versus RHE either visually (e.g., bubble formation) or from electrochemical data (Figure 5). The obtained current density is attributed exclusively to the ongoing oxidation/dissolution processes. Similarly, Mo_2_NiB_2_ also did not show any OER activity in the applied potential range (Figure 5), and the observed current density can be attributed to the oxidation processes, accompanied by the dissolution of molybdenum and boron. Contrary to Mo_2_ *TM*B_2_ (*TM* = Fe and Ni), CVs with the Mo_2_CoB_2_ electrode possess a lower offset current density and clear OER onset potential at 1.52 V versus RHE (Figure 5), being lower than those for all reference metals (Figure S11). Furthermore, significant current densities of 800–900 mA cm^–2^ can be achieved at the highest measured potential of 2 V versus RHE.

**Figure 5:**
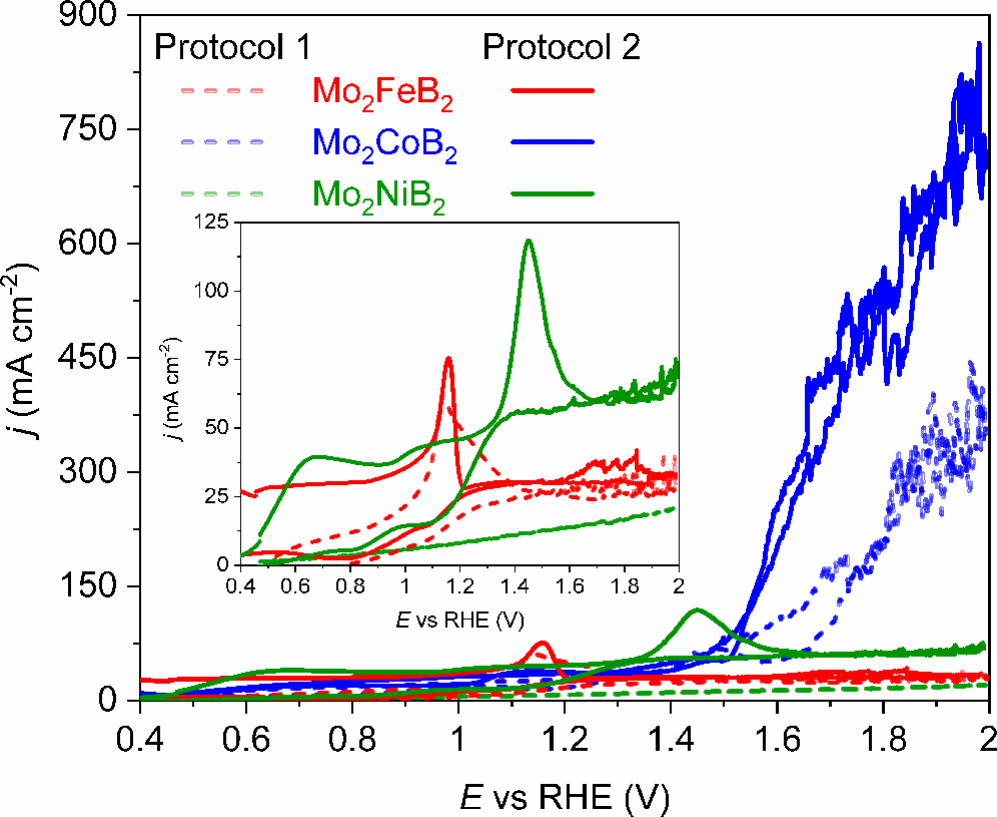
CVs with Mo_2_ *TM*B_2_ electrodes in the OER potential range after surface pretreatment according to protocols 1 (short dash lines) and 2 (solid lines). For protocols, see the Experimental Section and SI. Inset: magnification of CVs for Mo_2_FeB_2_ and Mo_2_NiB_2_ (*j* = 0–125 mA cm^–2^).

Last but not least, among the reference metals, elemental Ni demonstrates the lowest onset potential of the OER among *TM* while the highest current densities at the maximum measured potential (2 V versus RHE) were obtained with the Co anode (Figure S11). Still, the OER onset potential with the Mo_2_CoB_2_ electrode is the lowest among all of the materials studied.

Comparing the electrochemical data after different pretreatments, Mo_2_FeB_2_ showed almost the same CV features, corresponding to the Mo^3+^/Mo^4+^ transition, but no OER activity (Figures 5 and S12a). The similar chemical behavior of Mo_2_FeB_2_ during pretreatment using different protocols, as well as the OER inactivity of this compound, is probably due to the coverage of active species by the *in situ*-formed oxide layer with suppressed electrical conductivity. In case of Mo_2_CoB_2_ a noticeable offset current density at lower potentials and a promising OER activity with respect to elemental cobalt in the OER region were observed after pre-treatment with both CV protocols (Figures 5 and S12b). Contrary to Mo_2_ *TM*B_2_ (*TM* = Fe, Co), Mo_2_NiB_2_ illustrated a drastically different behavior in the OER region with the change of pretreatment procedures (Figures 5 and S12c). Significant OER activity with intense bubble formation was observed during the first CV cycle (Figure S12c). The onset potential of the OER of ∼1.59 V versus RHE is close to that for elemental Ni (Figure S11d). No oxygen evolution was observed in the successive CV cycles, only a significant offset current density and an oxidation peak placed at 1.45 V versus RHE.

Therefore, the oxygen evolution observed in the first scan is attributed to the altered pretreatment history of Mo_2_NiB_2_, facilitating surface saturation with electroactive species (e.g., nickel (oxy)­hydroxides stabilized by the residual molybdenum) rather than the dominance of anodic molybdenum dissolution.^8^

Due to the remarkable OER activity of Mo_2_CoB_2_ after both pretreatments, a stability measurement (CP) was performed at an elevated current density of 200 mA cm^–2^ (Figure S13). Similar oxidation features during CV pretreatment as well as comparable OER activity were observed before starting the CP experiment (Figure S13a,b), revealing the reproducibility of the electrochemical results from experiment to experiment (Figures 4a and 5). The stable values of measured potentials (ca. 1.8 V versus RHE) reveal the stability of OER activity of Mo_2_CoB_2_ during the initial 45 min of the CP experiment, which changes by their slow increase for next 25 min and, finally, a rapid increase of potential after 70 min of CP (Figure S13c). Such limited OER activity is due to the continuous thickening of the *in situ* oxide layers, making electron transfer in the electrical circuit difficult. This example illustrates once more the immense influence of the applied conditions as well as pretreatment procedures on the OER activity of any electrocatalyst. Broadly applied protocols with activity measurements in a limited potential range, short CP experiments at a low current density of 10 mA cm^–2^, and various surface modifications via electrochemical pretreatment can hide the chemical behavior and catalytic performance of these materials at real electrolysis conditions.

Due to the extremely oxidative conditions of the OER, inevitable structural transformations take place on the exposed surface, near the surface, or even in bulk regions of the electrodes, depending on their crystal structure and the chemical interactions between the components. Therefore, postcharacterization of the investigated electrode materials is an essential part of such studies. Since materials pretreated with different protocols reveal similar behavior in the OER region, only the characterization of the samples after pretreatment with protocol 1 and following OER experiments is presented here. Light microscopy of Mo_2_ *TM*B_2_ electrodes after OER experiments illustrates the surface inhomogeneity (particularly in the case of Mo_2_FeB_2_ and Mo_2_CoB_2_) as well as the appearance of differently colored regions (sometimes grains), revealing the presence of different oxidized species on the electrochemically exposed area (Figure S14). Contrary to Mo_2_ *TM*B_2_, the surfaces of the reference Fe, Co, and Ni remain smooth and homogeneous, and changes after EC were hardly visible (Figure S15). XRD analysis in reflection mode demonstrates the presence of predominantly Mo_2_ *TM*B_2_ phases (Figure S16). Moreover, Mo_3_ *TM*B_3_ secondary phases were still distinguishable in Co- and Ni-containing materials.

Surface oxidation either enhances the oxygen evolution in the case of optimal binding energies for the reaction intermediates or leads to material dissolution, which is a degradation process that may lead to bulk material instability under harsh electrochemical conditions.^4,86,87^ The morphological changes of Mo_2_ *TM*B_2_ under the OER conditions, obvious from light microscopy, necessitate further analysis of these materials by scanning electron microscopy (SEM) coupled with energy-dispersive X-ray spectroscopy (EDXS). The electrochemically exposed areas of Mo_2_ *TM*B_2_ are inhomogeneous and rough and possess regions with different SEM contrast (Figure 6). To attribute or exclude the compositional differences causing such contrast, the metal contents (at. %) at different regions of the EC-exposed areas were compared. The elemental mapping clearly pointed out the large regions with significant depletion of Mo content due to its oxidation and accompanying leaching under the OER conditions. The simultaneous enrichment in *TM*, K, and O was obvious in the Mo-leached regions, resulting in respective deviations of Mo/*TM* ratios from 2 (Figure 6, right panel). In the Mo-depleted regions, its content drops down to about 10 at. % compared to 67 at. % in the pristine materials (due to the low atomic weight of boron, its quantitative EDXS analysis was omitted and only Mo and *TM* contents were estimated). The Mo/*TM* ratios in other regions (the light gray areas on BSE images in Figure 6a,b) are higher than the ratio on the Mo-leached regions, showing that the material after partial detachment of the *in situ*-formed layers still have significant amount of molybdenum.

**Figure 6:**
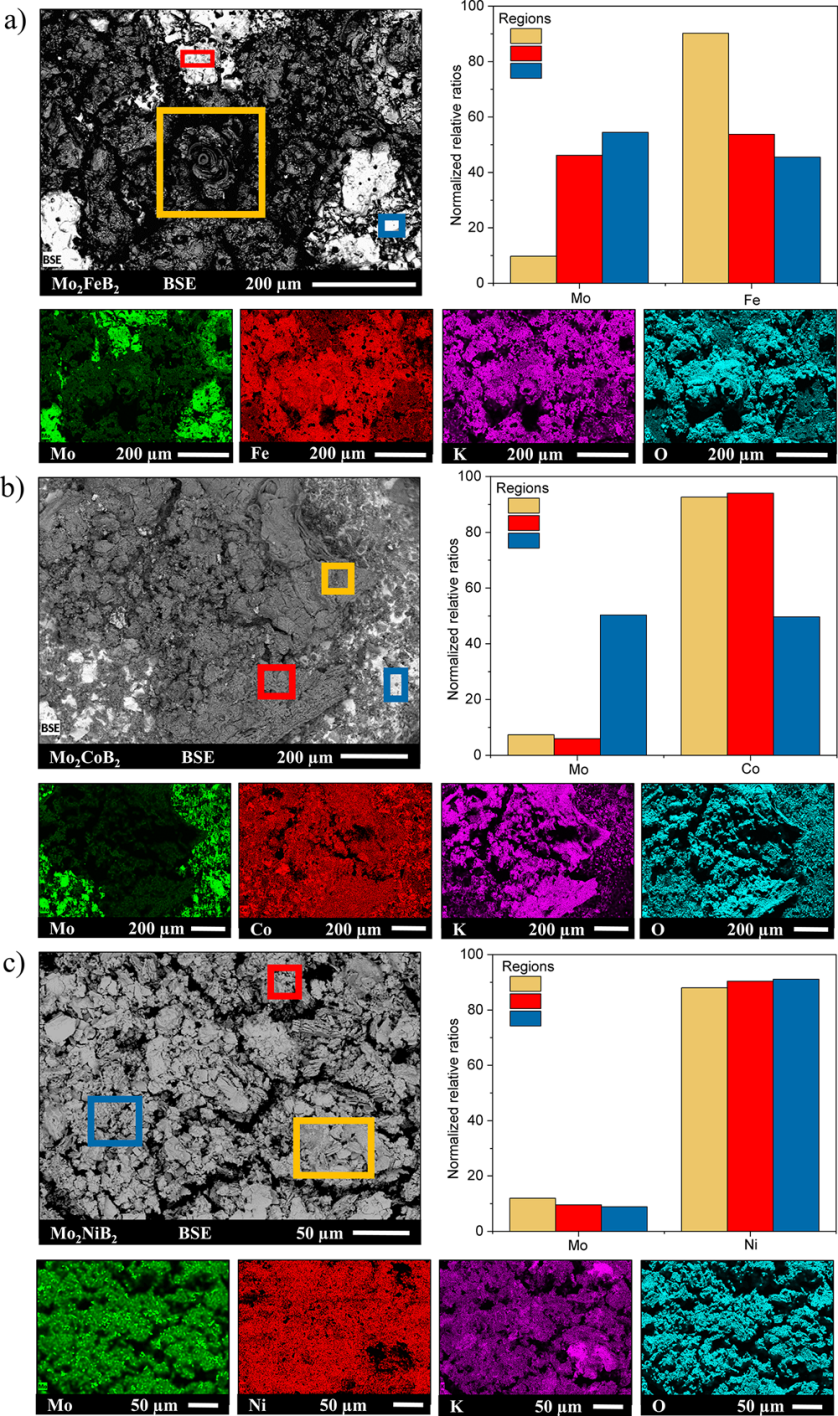
BSE images of Mo_2_ *TM*B_2_ (*TM* = Fe (a), Co (b), and Ni (c)) electrodes after the OER (left panel), supported by elemental mappings. Bar graphs represent normalized relative ratios between Mo and *TM* in different regions of the spent electrodes (right panel).

The active anodic dissolution of molybdenum and boron, irrespective of the protocol used for electrode pretreatment, is also evident from the elemental analysis of the spent electrolytes using inductively coupled plasma-optical emission spectroscopy (ICP-OES) (Table 2). The amounts of *TM* in the electrolyte after OER experiments are below the detection limit of the ICP-OES method (<0.1 mg L^–1^). The concentrations of molybdenum and boron in the electrolyte after experiments with Mo_2_NiB_2_ are lower than those with Mo_2_FeB_2_ and Mo_2_CoB_2_. It is known that electrochemical oxidation initiates the anodic metal dissolution, meaning migration of the metal ions from the surface to the electrolyte.^88^ At high pH values (alkaline media) and applied potentials above 1.2 V versus RHE, molybdenum and boron are found in the electrolyte in the form of soluble molybdate and borate, whereas iron, nickel, and cobalt are present in the oxide form on the electrode.^82^

**Table 2:** Concentrations of Mo and B (in mg L^–1^) in Electrolytes after the OER Experiments (ICP-OES Data)

	**protocol 1**		protocol 2	
**electrode**	**[Mo]**	**[B]**	**[Mo]**	**[B]**
**Mo**_**2**_ **FeB**_**2**_	50.2 (7)	7.1 (1)	49.8 (1)	5.8 (0)
**Mo**_**2**_ **CoB**_**2**_				
standard OER	39.8 (8)	5.3 (1)	42.6 (1)	5.5 (0)
long-term CP			75.7 (1)	10 (0)
**Mo**_**2**_ **NiB**_**2**_	26.8 (0)	3.1 (0)	36.2 (1)	4.6 (0)

This explains the high content of molybdenum and boron in the electrolyte evidenced by ICP-OES. Furthermore, almost double amounts of molybdenum and boron were found in KOH after the CP measurement with Mo_2_CoB_2_ at 200 mA cm^–2^ (Table 2), demonstrating the continuous leaching of these components during the CP. Similar to the short OER experiment, no Co was detected after 70 min of the CP experiment at elevated current density.

*Ex situ* XPS measurements provide information about the surface and near-surface regions of the electrodes after electrochemical experiments. To illustrate the changes in the chemical states of *TM*, the XP spectra of their 2*p* core levels are presented in Figure 7. Due to the strong charging effect on the oxidized surfaces of the OER-exposed electrodes, the peak positions were aligned with respect to the corresponding C 1*s* binding energy measured for each pristine Mo_2_ *TM*B_2_ sample. First of all, the shift of the *TM* 2*p* core levels to higher binding energies and broadening of the peaks, as well as the shift of the onset of the VB (indicating change from metallic to less conductive state (Figure S17a)), indicate the formation of oxide/hydroxide layers on the surfaces of Mo_2_ *TM*B_2_ under a harsh oxidative reaction environment. XP spectra of the Mo_2_FeB_2_ electrode clearly show complete oxidation of the surface with Fe_2_O_3_ (Fe 2*p*_3/2_: 711.9 eV and Fe 2*p*_1/2_: 725.4 eV), MoO_3_ (Mo 3*d*_5/2_: 233.5 eV and Mo 3*d*_3/2_: 236.6 eV), MoO*_*x*_* (*x* = 2–3, Mo 3*d*_5/2_: 231.4 eV), and B_2_O_3_ (B 1*s*: 193.4 eV) formed on it (Figure 7). The binding energies of Fe 2*p*, Mo 3*d*, and B 1*s* core level peaks related to the oxidized part of molybdenum, iron, and boron (Figure 7), respectively, are close to those found in the literature.^76,89–92^ The corresponding XP spectra of the O 1*s* core level confirm the presence of the above-mentioned oxides on the surface of the OER-exposed Mo_2_FeB_2_ (Figure S17b). The dominance of Fe_2_O_3_ on the surface of Mo_2_FeB_2_ from the *ex situ* XPS measurement and the OER inactivity of Mo_2_FeB_2_ suggest the presence of inactive Fe_2_O_3_ species (with poor electrical conductivity) on the surface also during the reaction.

**Figure 7:**
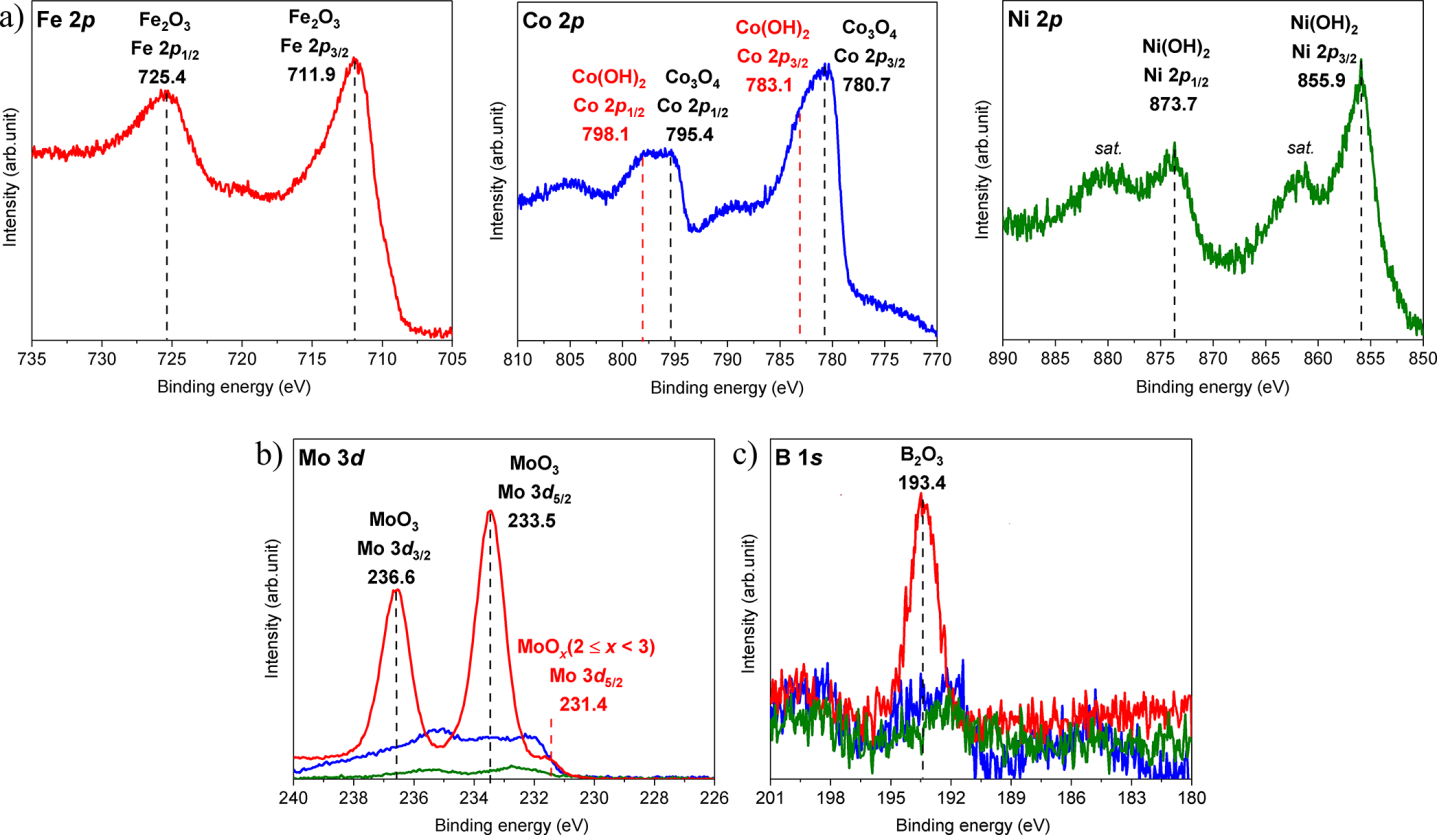
XP *TM* 2*p* (a), Mo 3*d* (b), and B 1*s* (c) core level spectra for Mo_2_ *TM*B_2_ electrodes after OER (colored). Dashed lines and respective numerical values represent the binding energies for the oxide and hydroxide species.

Significantly altered surface after surface pre-treatment and OER was also evidenced for the Mo_2_CoB_2_ electrode (Figure 7a). The sharp signal, corresponding to the intermetallic Co in pristine Mo_2_CoB_2_ (Figure 3a), was completely diminished after electrochemical treatment. Instead, broad peaks appear in the region of cobalt oxide/hydroxides, e.g., Co­(OH)_2_ and Co_3_O_4_ (Figure 7a). It is reasonable to consider the dominance of Co_3_O_4_ (with Co 2*p*_3/2_: 780.7 eV and 2*p*_1/2_: 795.4 eV peaks, in line with the literature^93^) on the surface under strong oxidation reaction conditions. On the other hand, Co­(OH)_2_ (with Co 2*p*_3/2_: 783.1 eV and 2*p*_1/2_: 798.1 eV peaks being in accord with ref 94; Figure 7a) may be formed upon partial reduction of highly oxidized cobalt species (OER active) with the release of applied potential. Furthermore, the incomplete transition of active cobalt oxide species to cobalt hydroxide with a limited reaction time could be another reason for such a broad peak. Also, the broad O 1*s* peak after OER indicates not only contributions from Co_3_O_4_ but also from Co­(OH)_2_ (Figure S17c). Contrary to the obvious Co oxide contribution, XP spectra of Mo 3*d* and B 1*s* core levels did not show any distinctive peaks at binding energies characteristic for the intermetallic contribution, and only very low in intensity, broad, and almost indistinguishable signals at binding energies, corresponding to MoO_3_ and B_2_O_3_. This is possibly due to a minor precipitation of solvated molybdate and borate ions by the time of electrode removal (Figure 7b,c).

The partial Mo and B oxidation and following dissolution from the surface of the Mo_2_NiB_2_ electrode presumably hinders the formation of OER-active nickel oxides/(oxy)­hydroxides such as Ni_2_O_3_, NiOOH, etc. Instead of that, nickel hydroxide forms as a passivation layer. The broad peaks on the XP spectra of Ni 2*p* core levels (2*p*_3/2_: 855.9 eV, 2*p*_1/2_: 873.7 eV) together with characteristic satellites represent the dominance of Ni­(OH)_2_ (Figures 7a and S17d), being in good agreement with available XPS studies on nickel hydroxide.^95,96^ Here, the signals on XP Mo 3*d* and B 1*s* spectra were not distinguishable from the background (Figure 7b,c). As the *in situ*-formed Ni oxidized layers were not detached and covered the complete electrochemically exposed area of the Mo_2_NiB_2_ electrode, molybdenum and boron were not detected via XPS, in contrast to Mo_2_FeB_2_ and Mo_2_CoB_2_ electrodes, which had a considerable part of the electrode, from which *in situ*-formed layers were detached (Figure S14).

Summarizing, postcharacterization of Mo_2_ *TM*B_2_ materials exposed to severe reaction conditions of the OER shows that the bulk structure of these materials was preserved (XRD and SEM); however, the uppermost layers and near-surface areas underwent irreversible surface oxidation and reorganization (SEM, XPS, and ICP-OES). Pronounced oxidation and dissolution of Mo and B in the form of molybdate and borate lead to the formation of *TM* oxide/hydroxides on the surface. Their nature, crystallinity degree, amount, and homogeneity of distribution are strongly dependent on the degree and rate of Mo and B dissolution. In terms of the OER activity, Mo_2_CoB_2_ possesses noticeable OER activity, outperforming the OER activity of elemental Co.

## Conclusions

The (electro)­chemical behavior of the intermetallic compounds Mo_2_ *TM*B_2_ (*TM* = Fe, Co, Ni) under oxygen evolution reaction conditions in an alkaline electrolyte has been systematically studied. Considering the harsh oxidative conditions of OER, chemical changes of the electrode materials are decisive for the OER activity data interpretation. In the case of Mo_2_ *TM*B_2_ electrodes, electrocatalytic performance is strongly dependent on the degree and pathway of the surface oxidation and following partial dissolution of Mo and B. In the case of Mo_2_FeB_2_, partial oxidation with formation of MoO_3_, Fe_2_O_3_, and B_2_O_3_ on the surface has already been demonstrated for the pristine material (due to the exposure to air), and OER experiments only deepen this process leading to even deeper dissolution of Mo and B with simultaneous formation of Fe_2_O_3_ layer with limited electrical conductivity and consequently inactivity for OER. Chemical behavior of Mo_2_NiB_2_ varies depending on the applied protocol, but severe molybdenum and boron dissolution also results in continuing material degradation during the application of anodic potential. In contrast, Mo_2_CoB_2_ possesses outstanding OER activity (compared to elemental Co) due to the interplay of different Co compounds on the dynamic *in situ*-formed surface.

## Supplementary Material



## Notes

Electrochemical procedures and setup; characterization of pristine materials (experiments and quantum chemical calculations); CV pretreatment of Mo_2_ *TM*B_2_ (*TM* = Fe, Co, Ni) and reference metals according to different protocols; OER activity of studied materials compared to reference metals; stability of the OER activity of the Mo_2_CoB_2_ electrode; and bulk- and surface-sensitive postcharacterization of Mo_2_TMB_2_ electrodes after electrochemical experiments (PDF)

## Abbreviations

arb. units: arbitrary units
*TM*: transition metal
OER: oxygen evolution reaction
SPS: spark plasma sintering
PXRD: powder X-ray diffraction
DSC: differential scanning calorimetry
EC: electrochemical
LM: light microscopy
SEM: scanning electron microscopy
EDXS: energy-dispersive X-ray spectroscopy
WDXS: wavelength-dispersive X-ray spectroscopy
BSE: backscattered electron
SE: secondary electron
XPS: X-ray photoemission spectroscopy
PEEK: polyetheretherketon
RHE: reference hydrogen electrode
OCV: open-circuit voltage
LSV: linear sweep voltammetry
CV: cyclic voltammetry
CP: chronopotentiometry
ICP-OES: inductively coupled plasma-optical emission spectrometry
FPLO: all-electron full-potential local orbital method
LDA/LSDA: local (spin) density approximation
DOS: density of states
pDOS: projected density of states
ΔSCF: delta self-consistent field
ED: electron density
ELI-D: electron localizability indicator
QTAIM: quantum theory of atoms in molecules
