# Supplementary material for: Chemical Behavior of Mo2 TMB2 (TM = Fe, Co, Ni) upon the Oxygen Evolution Reaction (OER)
Source: ACS Mater Au. 2025 Jun 24;5(4):718–31. doi: 10.1021/acsmaterialsau.5c00035 (PMC12257420; doi:10.1021/acsmaterialsau.5c00035)
Supplement: Supplementary file 1 [file mg5c00035_si_001.pdf]

Supporting Information

Chemical behavior of $Mo_2\mathit{TM}B_2$ (*TM* = Fe, Co, Ni) upon oxygen evolution reaction (OER)

Fatma Aras[a,*], Ulrich Burkhardt[a], Alim Ormeci[a], Horst Borrmann[a], Simone G. Altendorf[a], Yuri Grin[a], Iryna Antonyshyn[a,b,*]

[a]Max-Planck-Institut für Chemische Physik fester Stoffe, Nöthnitzer Str. 40, 01187 Dresden, Germany

[b]Fritz-Haber-Institut der Max-Planck-Gesellschaft, Faradayweg 4-6, 14195 Berlin, Germany

*Corresponding authors: Fatma.Aras@cpfs.mpg.de, Antonyshyn@fhi-berlin.mpg.de

Figure S1. Scheme and photo of the PEEK house-made three-electrode electrochemical cell (*a*) and the sequence of applied techniques for electrochemical measurements (*b*).

Figure S2. BSE images of as-synthesized Mo_2FeB_2 (*a*), Mo_2CoB_2 (*b*) and Mo_2NiB_2 (*c*) samples.

Figure S3. ELI-D bond basins in Mo_2NiB_2 (*a*), Mo_2CoB_2 (*b*) and Mo_2FeB_2 (*c*).

Figure S4. Chemical bonding in side-centered rhombic prisms $[(B_2)Co_4Mo_4Mo_4]$ in Mo_2CoB_2 (*a*) and $[(B_2)Mo_8Fe_4]$ in Mo_2FeB_2 (*b*) in form of contributing bond basins.

Figure S5. The XP O 1*s* core level spectra of as-synthesized Mo_2FeB_2 (*a*), Mo_2CoB_2 (*b*) and Mo_2NiB_2 (*c*) samples.

Figure S6. Comparison of the experimental XP valence bands (*colored curves*, normalized to 1) of pristine Mo_2TMB_2 (*a*), comparison of the XPS data (*colored curves*) with the spectra obtained from the electronic structure calculations (*black curves*) (*b-d*).

Figure S7. Total, atom and orbital projected DOS computed for Mo_2FeB_2, with antiferromagnetically coupled Fe magnetic moments (*a*), Mo_2CoB_2 (*b*), Mo_2NiB_2 (*c*).

Figure S8. CV pre-treatment of Mo_2TMB_2 (*TM* = Fe (*red, a*), Co (*blue, b*), Ni (*green, c*)) according protocol 1, represented with 50 cycle increase.

Figure S9. CVs of reference Mo (*a*), Fe (*b*), Co (*c*) and Ni (*d*), followed protocol 1 and represented with 50 cycle increase.

Figure S10. CV features of elemental Mo (protocol 2), represented with 5 cycle increase.

Figure S11. First and the last CV cycles with Mo electrode (*a*) in the OER region. CV features of Fe (*b*), Co (*c*), Ni (*d*) electrodes, pre-treated according to protocol 1 and 2, before and after chronopotentiometry (CP, j = 10 mA cm^{-2}, 2h).

Figure S12. Three CV cycles of Mo_2FeB_2 (*a*), Mo_2CoB_2 (*b*), and Mo_2NiB_2 (*c*) in the region of OER after pre-treatment according to protocol 2.

Figure S13. Stability of OER activity of Mo_2CoB_2 electrode: CV pre-treatment (*a*), OER activity before and after CP (*b*), CP at 200 mA cm^{-2} for 70 min (*c*).

Figure S14. Light microscopy (in bright field) of Mo_2TMB_2 (*TM* = Fe, Co, Ni) electrodes after OER experiments.

Figure S15. Light microscopy (in bright field) of Fe, Co, Ni and Mo foils after OER experiments.

Figure S16. XRD patterns (reflection mode) of Mo_2TMB_2 (*TM* = Fe (*a*), Co(*b*) and Ni(*c*)) electrodes before (*black*) and after OER (*colored*).

Figure S17. Valence band (*a*) and O 1*s* core level XP spectra of Mo_2FeB_2 (*red, b*), Mo_2CoB_2 (*blue, c*) and Mo_2NiB_2 (*green, d*) after OER (pre-treatment using protocol 1).

Table S1. Optimized atomic coordinates for Mo_2TMB_2 (*TM* = Fe, Co, Ni).

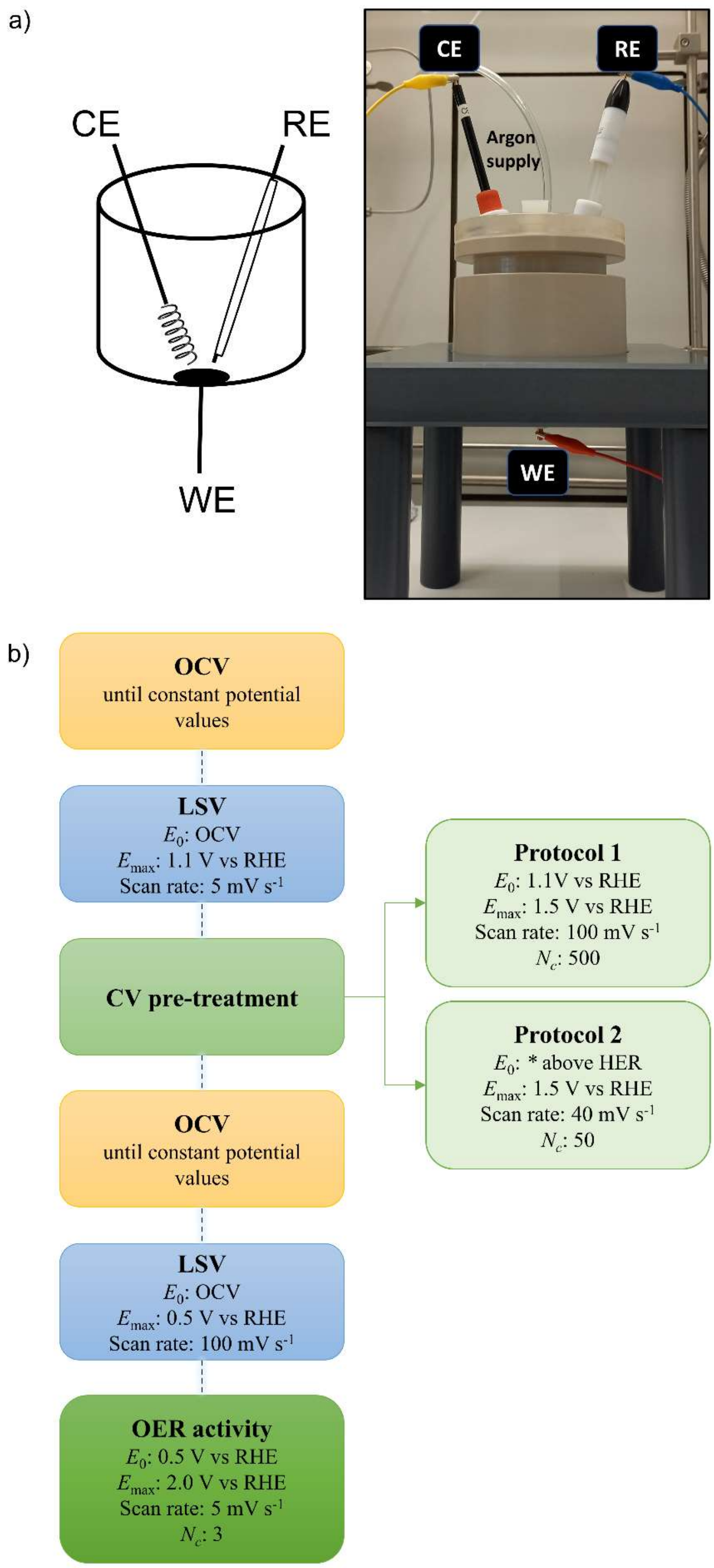

Figure S1. Scheme and photo of the PEEK house-made three-electrode electrochemical cell (*a*) and the sequences of applied techniques for electrochemical measurements (*b*).

Only in case of Mo_2CoB_2, a stability test at elevated current density of 200 mA cm^{-2} was performed using chronopotentiometry (CP) technique.

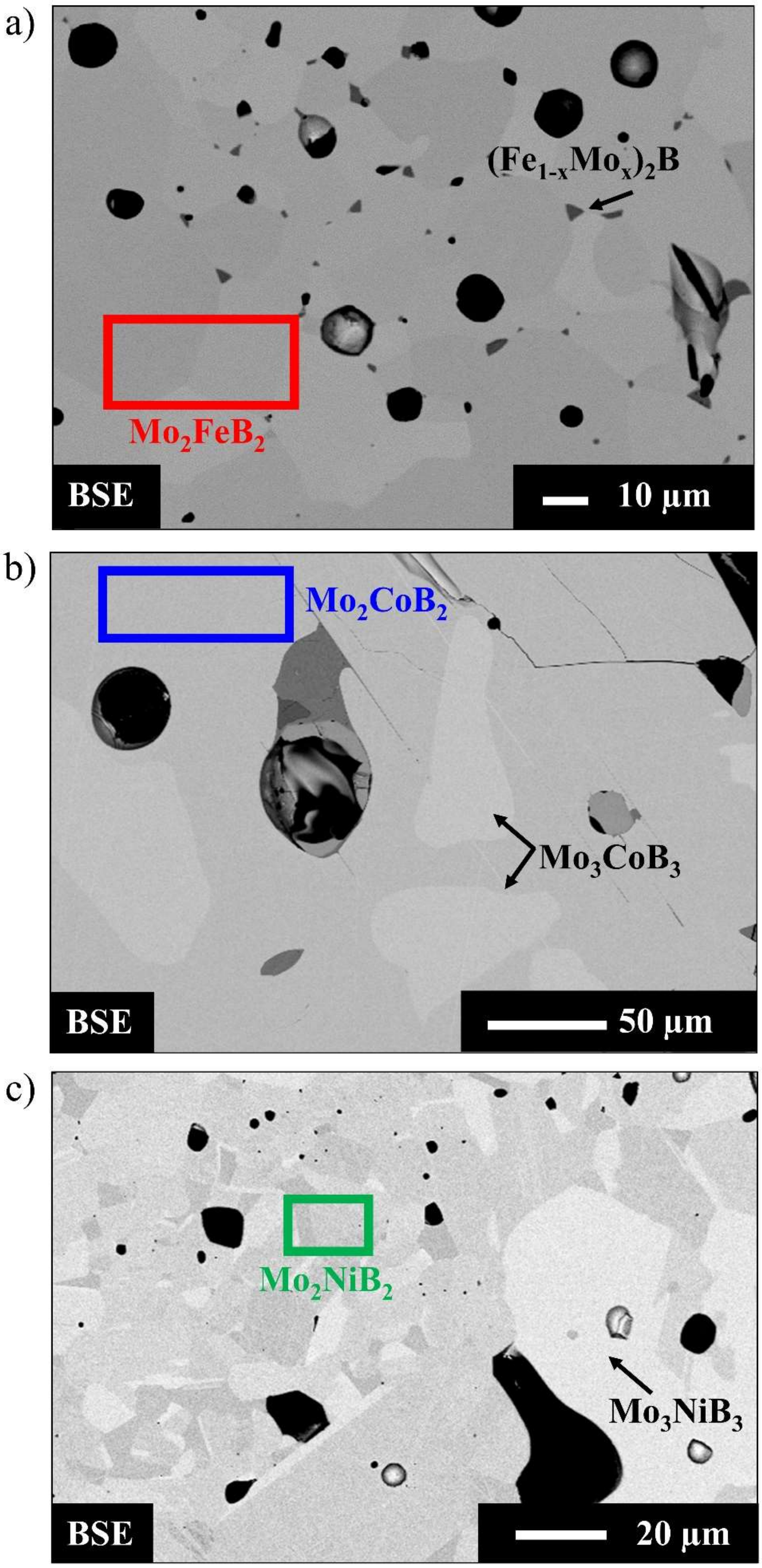

Figure S2. BSE images of as-synthesized Mo_2FeB_2 (*a*), Mo_2CoB_2 (*b*) and Mo_2NiB_2 (*c*) samples. The main matrices of Mo_2TMB_2 are highlighted by *colored rectangles*. The secondary phases are marked by *black arrows*.

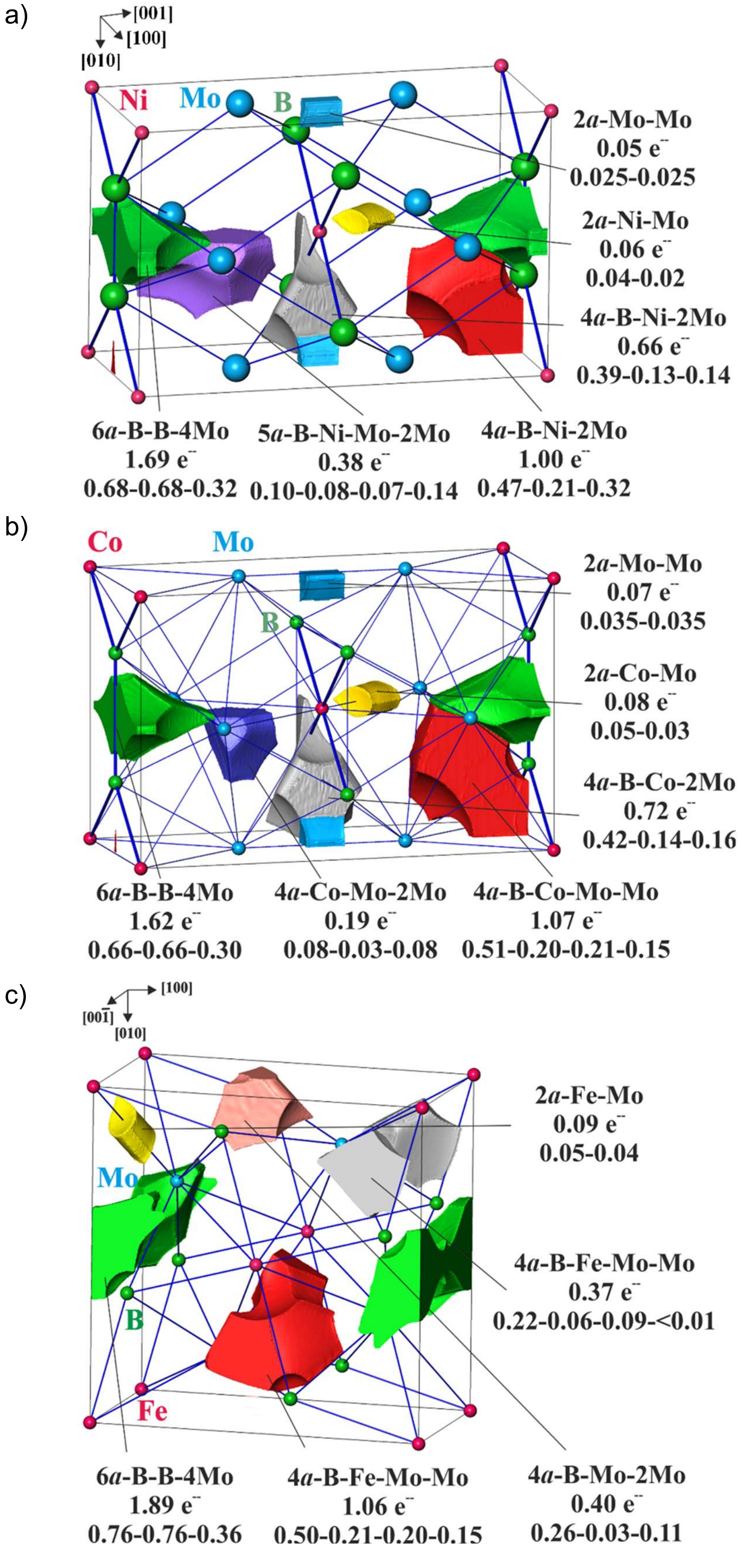

Figure S3. ELI-D bond basins in Mo_2NiB_2 (*a*), Mo_2CoB_2 (*b*) and Mo_2FeB_2 (*c*). For each basin, its atomicity (*a*) and participating atoms (*first line*), total basin population (*second line*) and individual contributions of the participating atoms (*third line*) are listed.

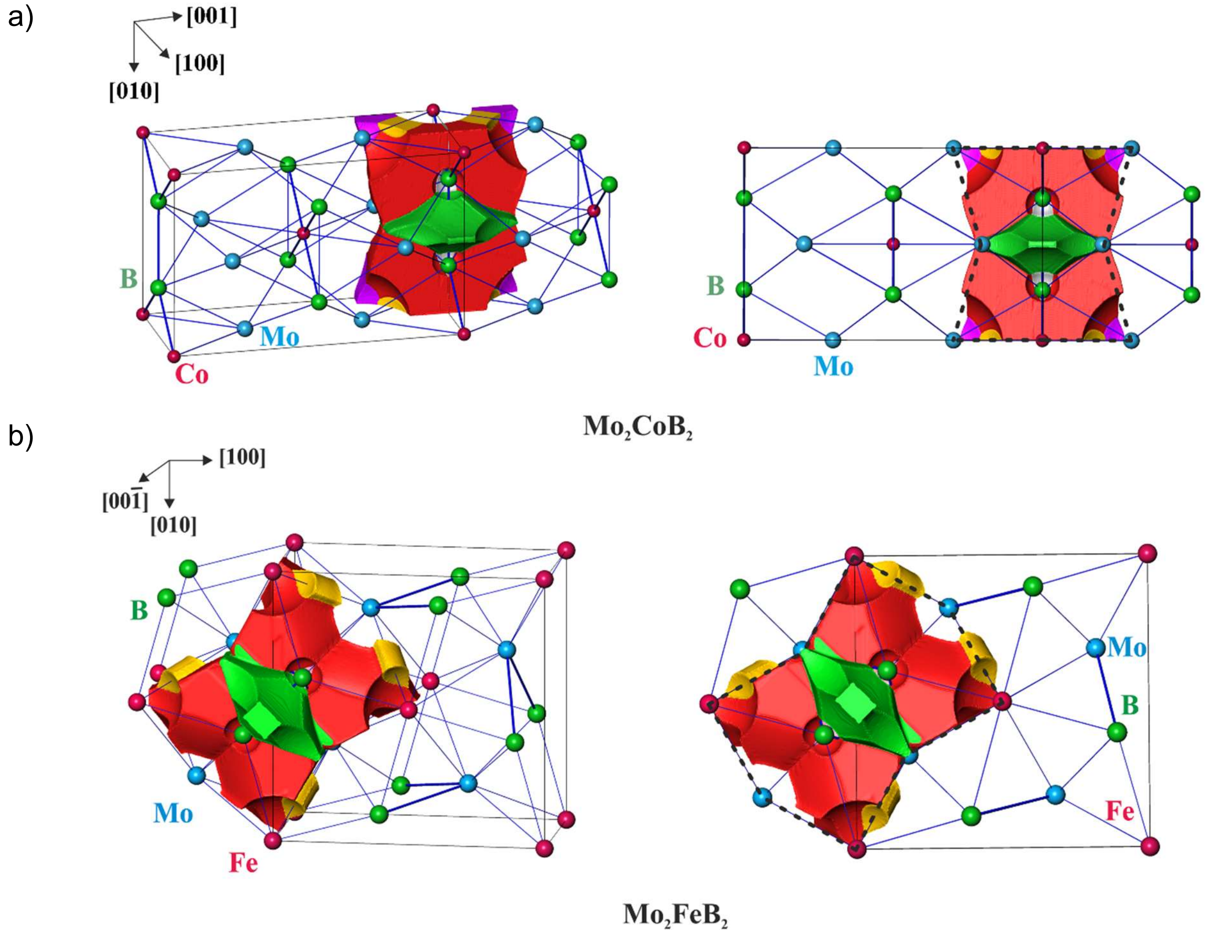

Figure S4. Chemical bonding in side-centered rhombic prisms $[(B_2)Co_4Mo_4Mo_4]$ in Mo_2CoB_2 (*a*) and $[(B_2)Mo_8Fe_4]$ in Mo_2FeB_2 (*b*) in form of contributing bond basins. The main part of the volume of side-centered rhombic prisms is formed by the basins of 6*a*-B-B-4Mo (*green*), 4*a*-B-*TM*-Mo-Mo (*red*) and 4*a*-B-Co-2Mo (*gray*) or 4a-B-Mo-2Mo (*light pink*) bonds (*left panel*). The low-populated basins of 2*a*-*TM*-Mo (*orange*) and 4*a* -Co-Mo-2Mo (*violet*) bonds are interconnecting the neighboring prisms (*cf. dashed black lines in the right panel*). The colors of the basins are the same as in Figure S3.

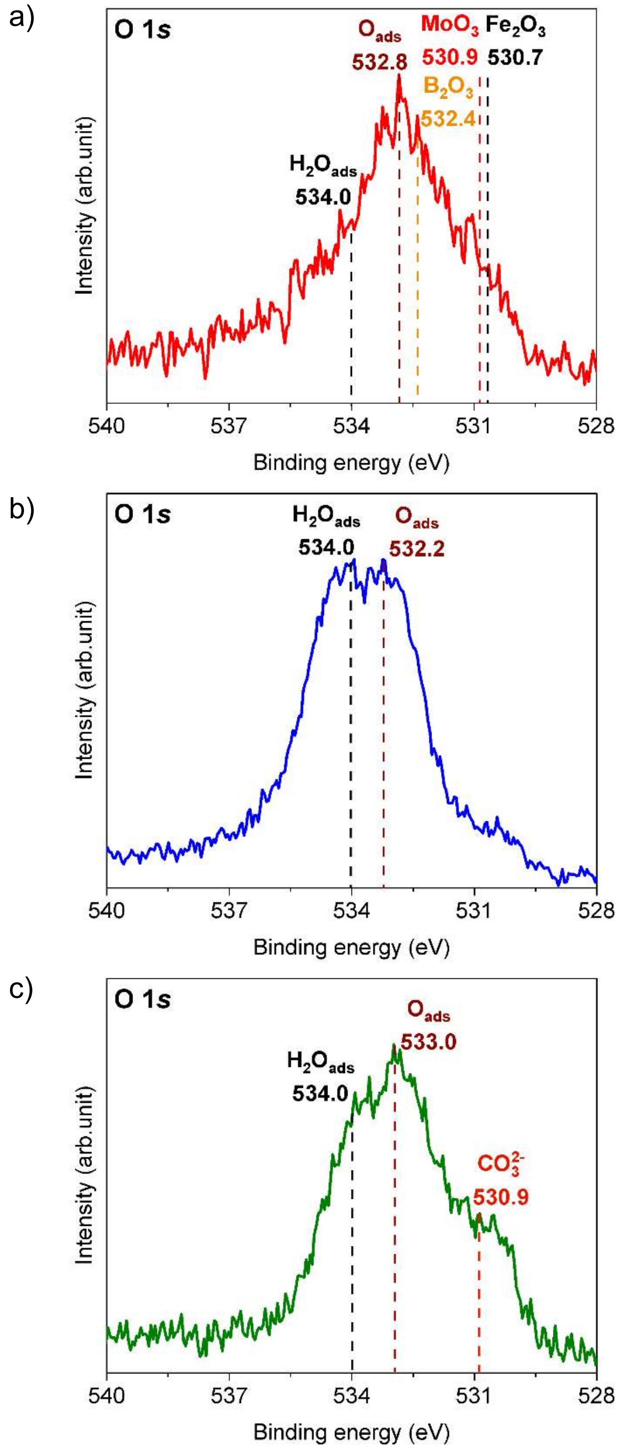

Figure S5. The XP O 1*s* core level spectra of as-synthesized Mo_2FeB_2 (*a*), Mo_2CoB_2 (*b*) and Mo_2NiB_2 (*c*) samples. The binding energies of adsorbed water (H_2O_{ads}), adsorbed O (O_{ads}), carbonate CO_3^{2-} as well as lattice oxygen in MoO_3, Fe_2O_3 and B_2O_3 are taken from Ref. 1-5.

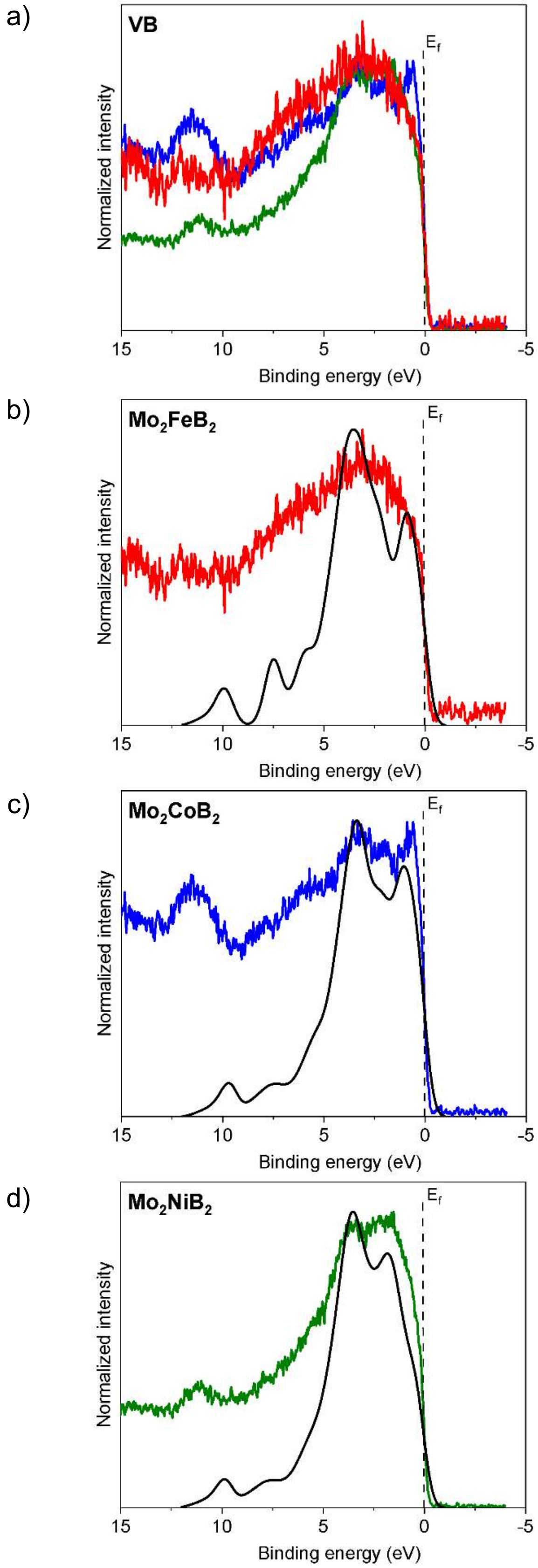

Figure S6. Comparison of the experimental XP valence bands (*colored curves*, normalized to 1) of pristine $Mo_2\mathit{TM}B_2$ (*a*), comparison of the XPS data (*colored curves*) with the spectra obtained from the electronic structure calculations (*black curves*) (*b-d*).

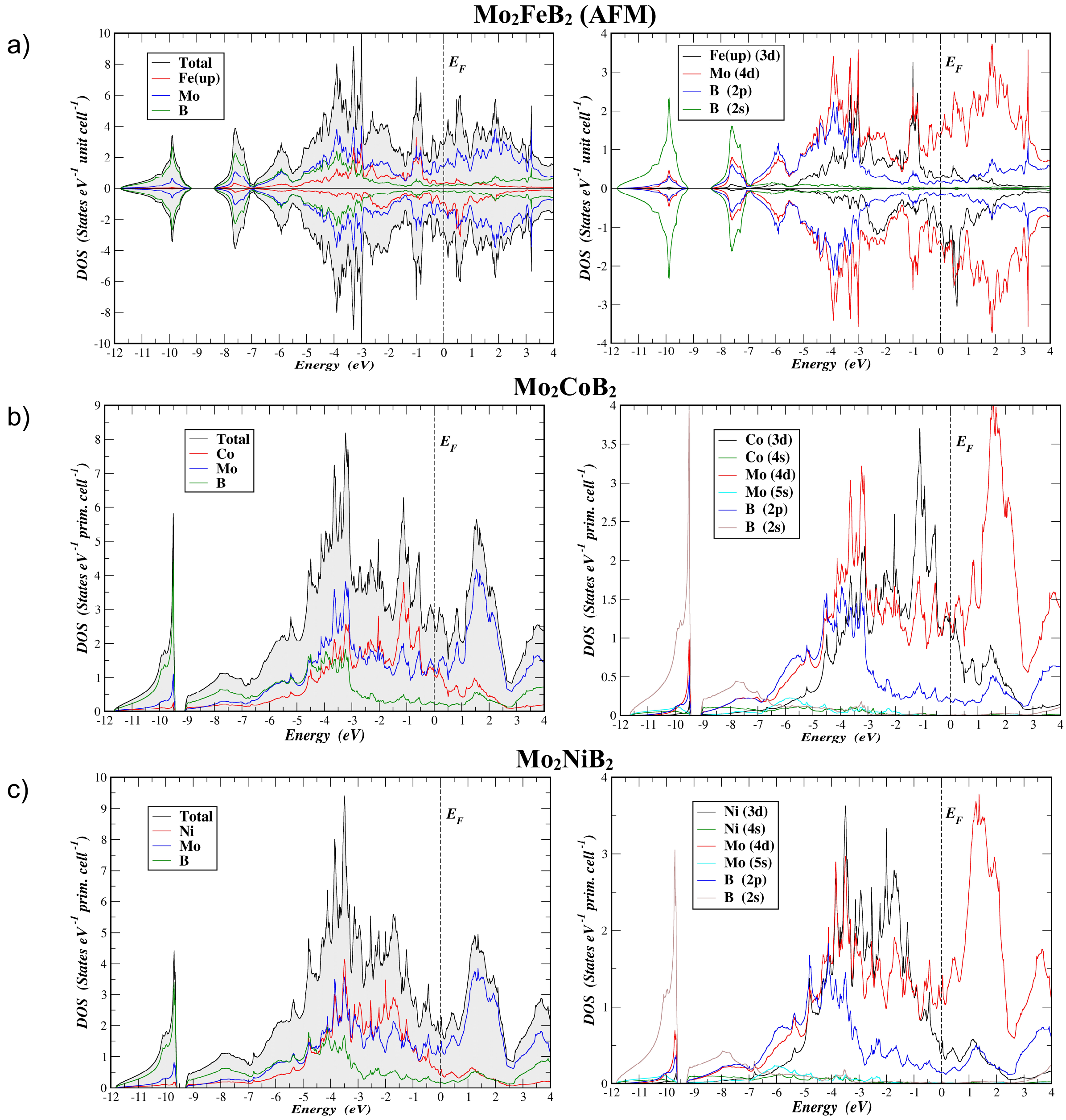

Figure S7. Total, atom and orbital projected DOS computed for Mo_2FeB_2, with antiferromagnetically coupled Fe magnetic moments (*a*), Mo_2CoB_2 (*b*), Mo_2NiB_2 (*c*).

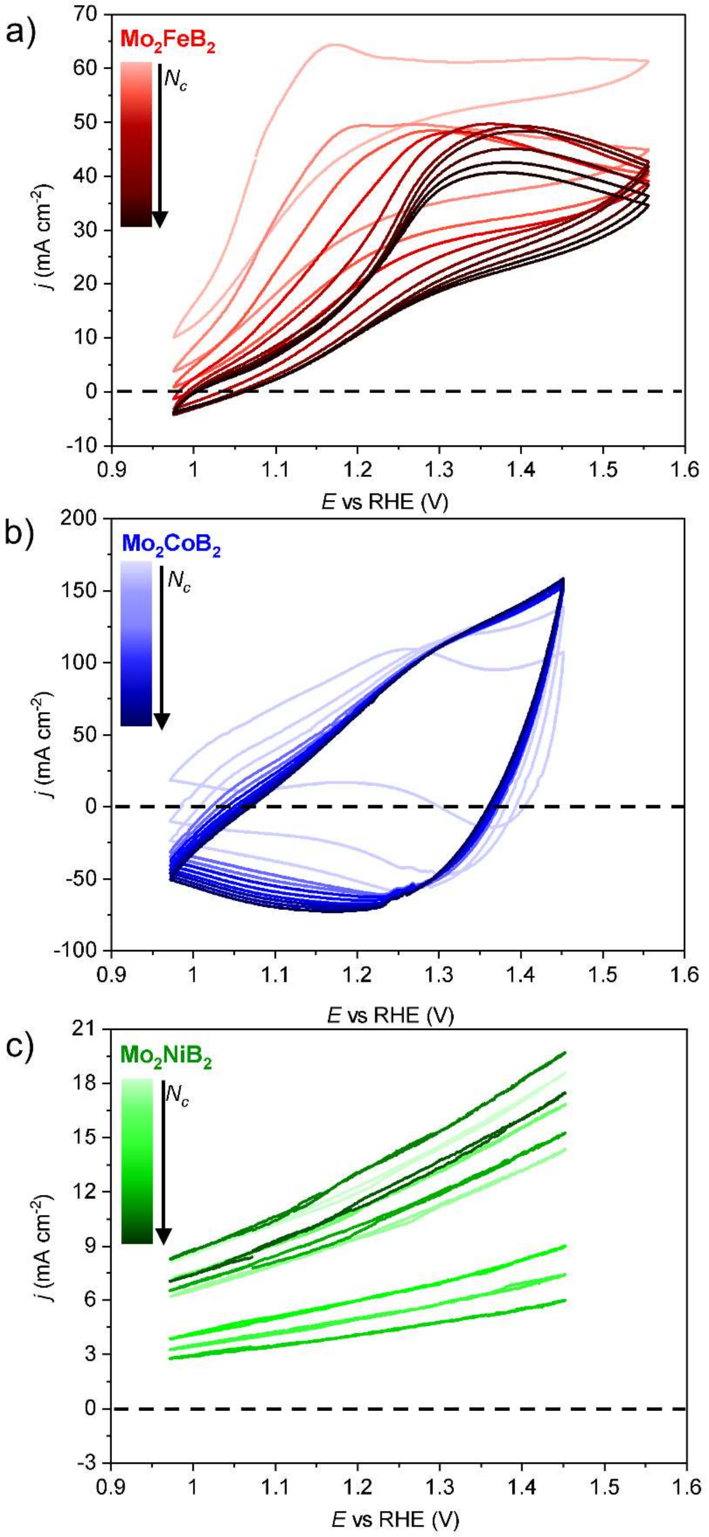

Figure S8. CV pre-treatment of Mo_2TMB_2 (*TM* = Fe (*red, a*), Co (*blue, b*), Ni (*green, c*)) according protocol 1, represented with 50 cycle increase. *Horizontal dashed line* guides the eye for distinguishing anodic and cathodic regions.

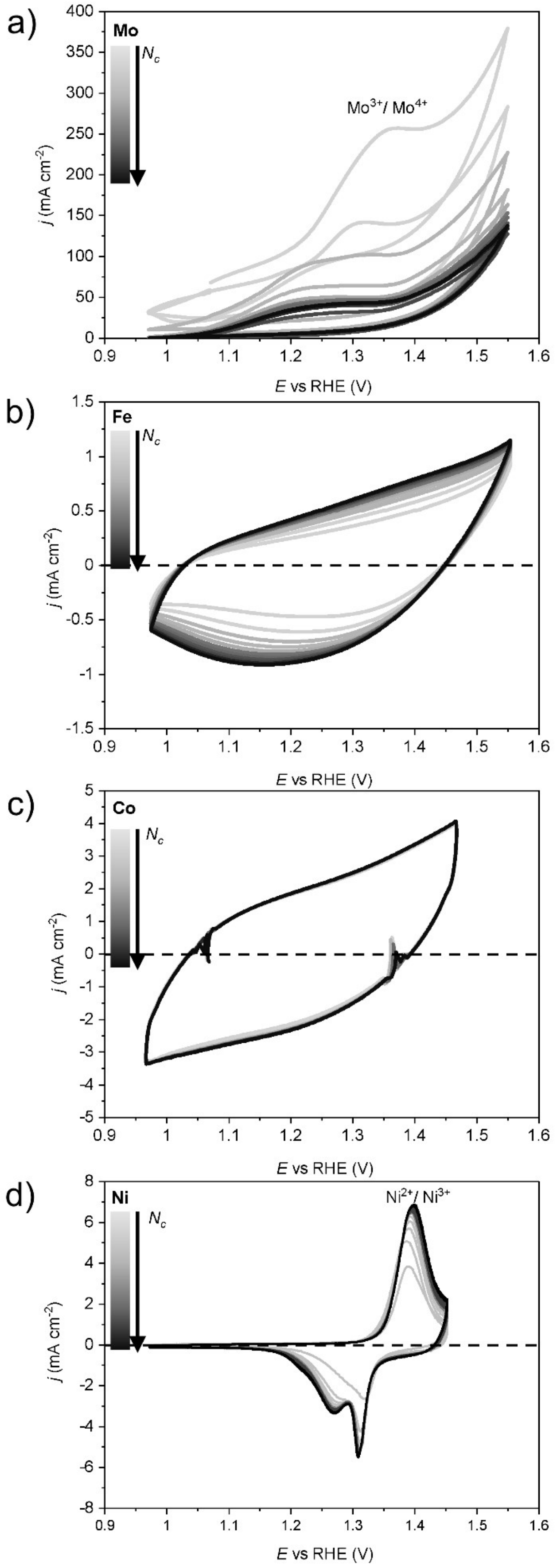

Figure S9. CVs of reference Mo (*a*), Fe (*b*), Co (*c*) and Ni (*d*), followed protocol 1 and represented with 50 cycle increase. *Horizontal dashed line* guides the eye for distinguishing anodic and cathodic regions.

To realize the differences in CV characteristics of transition metals (Fe, Co, Ni) and intermetallic compounds, the same reaction conditions (protocol 1) were applied to the reference materials. Elemental Fe did not show an oxidation peak in the potential range 1.0-1.5 V vs RHE, presumably, because of early formation of iron oxide/hydroxide films, which would then be observed as an oxidation peak in this potential range. The obtained current density is about 1 mA cm^{-2} and consists of both anodic and cathodic currents, resulting from the double capacitance (**Figure S9b**). Similar to Fe, no redox peaks for elemental cobalt were detected in the applied potential range (**Figure S9c**). On the other hand, the CV of elemental Ni possesses features that agree well with those reported in the literature (**Figure S9d**).[6] The oxidation peak at 1.39 V vs RHE, with a shift of only 0.1 V towards higher potentials upon cycling, corresponds to the formation of Ni oxyhydroxide from Ni hydroxide on the Ni surface.[6] The clear presence of reduction peaks points out the reversibility of this process.

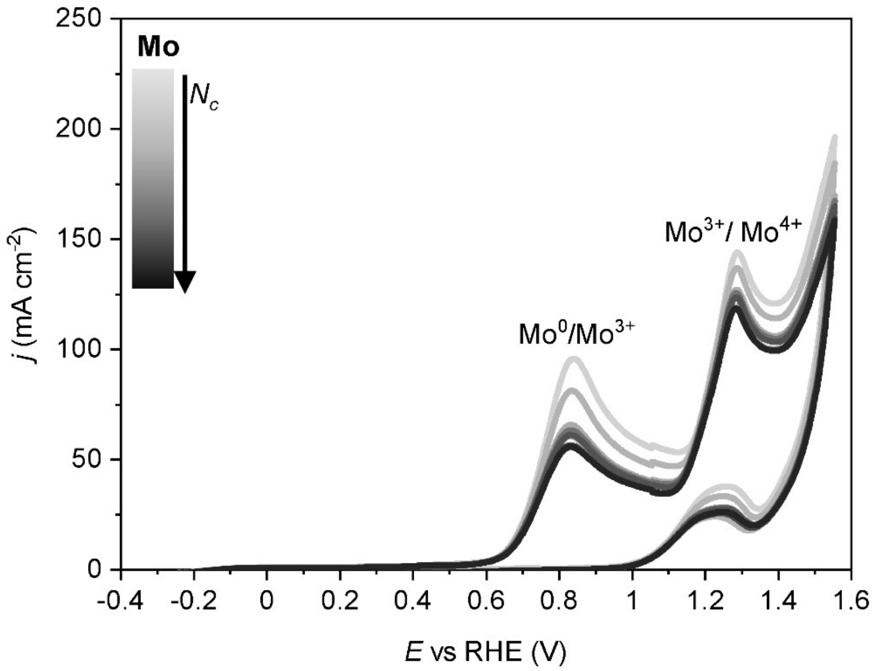

Figure S10. CV features of elemental Mo (protocol 2), represented with 5 cycle increase.

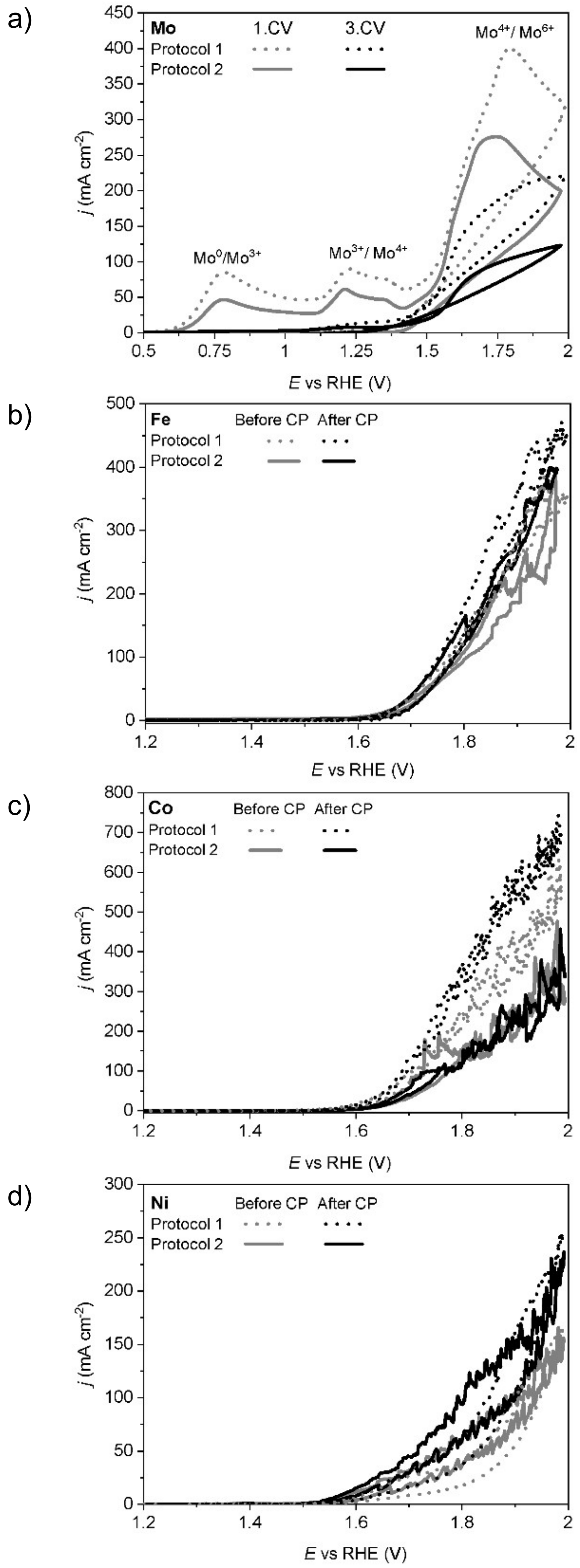

Figure S11. First and the last CV cycles with Mo electrode (*a*) in the OER region. CV features of Fe (*b*), Co (*c*), Ni (*d*) electrodes, pre-treated according to protocol 1 and 2, before and after chronopotentiometry (CP, j = 10 mA cm^{-2}, 2h). *CP for Mo was interrupted due to huge resistivity resulting from the severe corrosion.

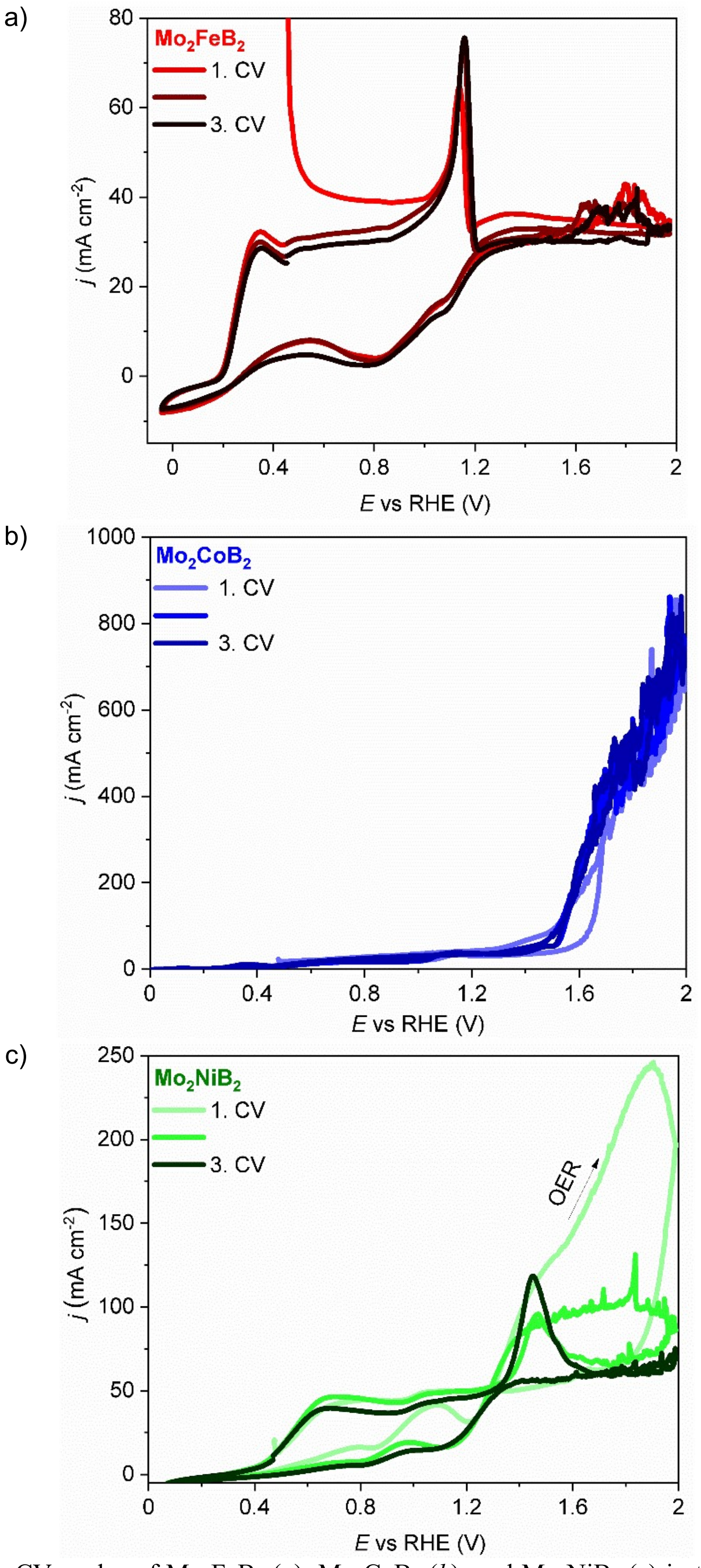

Figure S12. Three CV cycles of Mo_2FeB_2 (*a*), Mo_2CoB_2 (*b*), and Mo_2NiB_2 (*c*) in the region of OER after pre-treatment according to protocol 2.

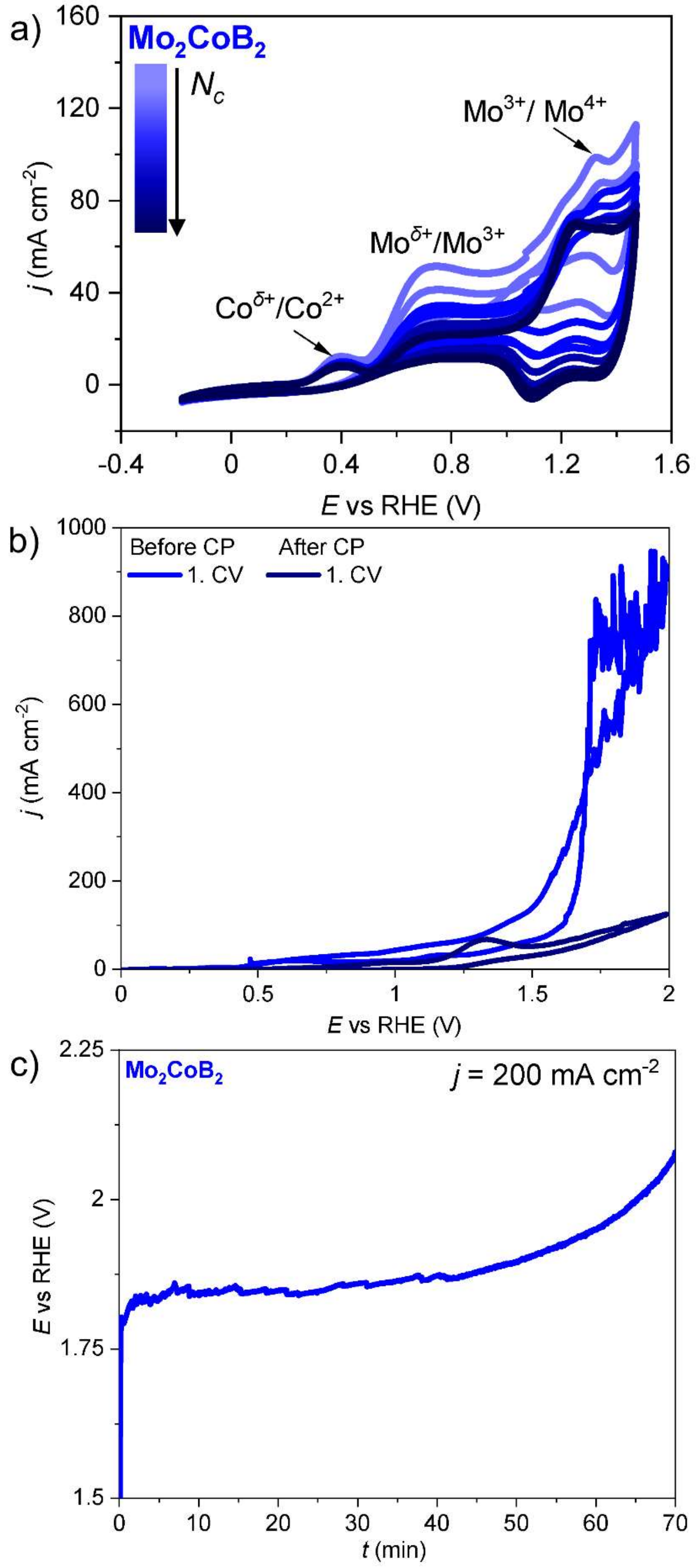

Figure S13. Stability of OER activity of Mo_2CoB_2 electrode: CV pre-treatment (*a*), OER activity before and after CP (*b*), CP at 200 mA cm^{-2} for 70 min (*c*).

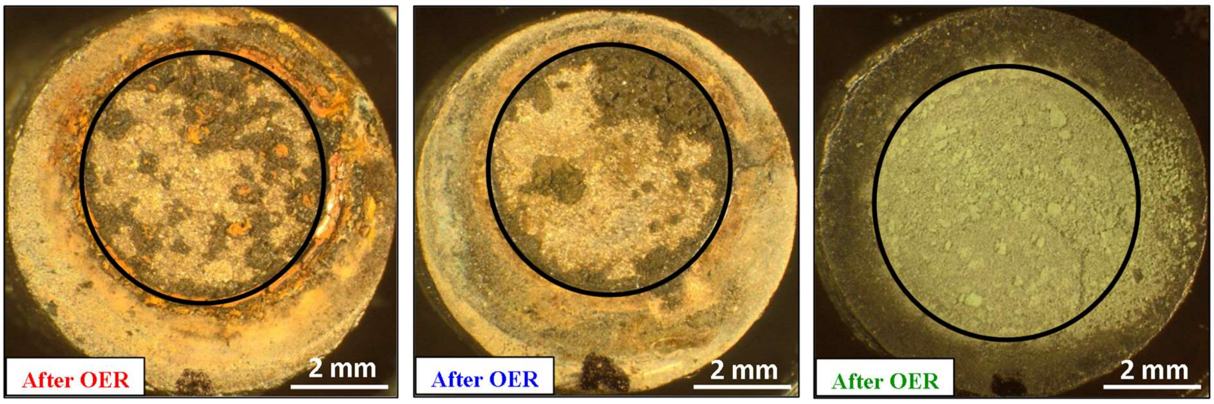

Figure S14. Light microscopy (in bright field) of $Mo_2\mathit{TM}B_2$ (*TM* = Fe, Co, Ni) electrodes after OER experiments. The electrochemically exposed areas are marked by the *black circles*.

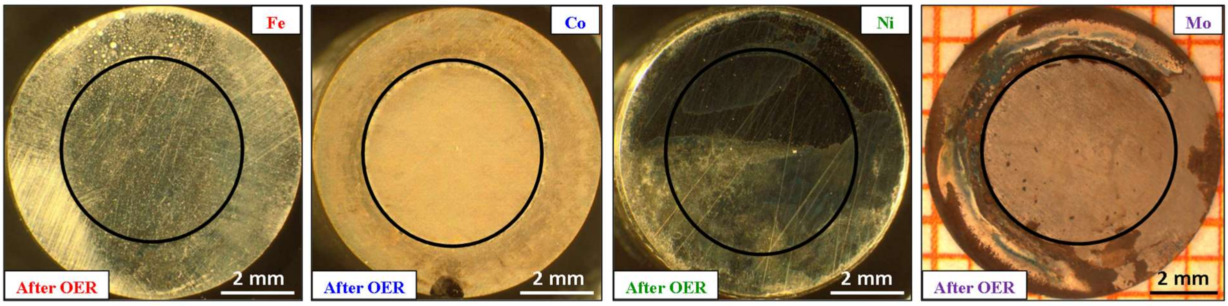

Figure S15. Light microscopy (in bright field) of Fe, Co, Ni and Mo foils after OER experiments. The electrochemically exposed area is marked by the *black circles*.

The molybdenum foil became black on the surface, in agreement with the strong oxidation of Mo according to the electrochemical data (*see above*).

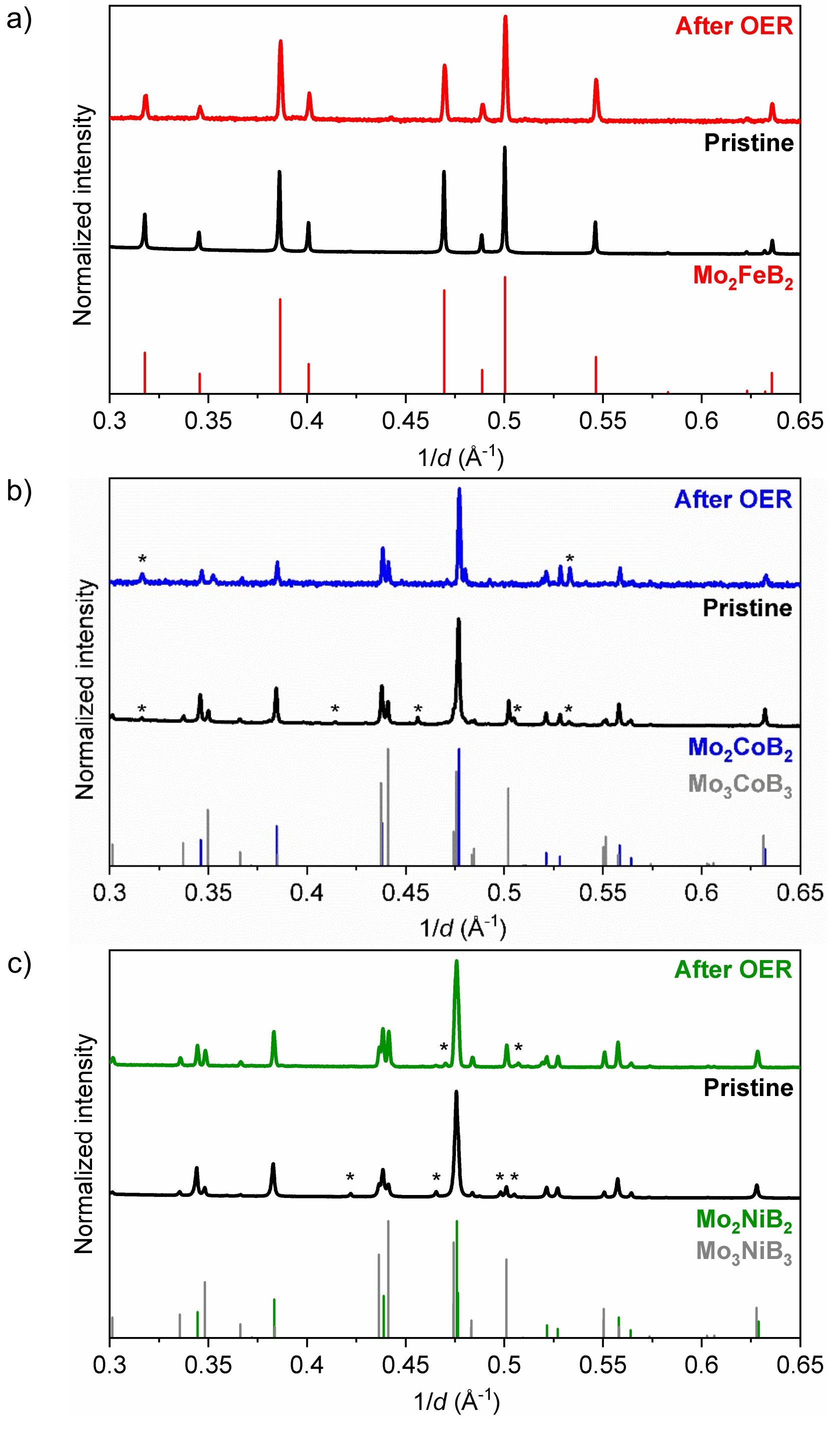

Figure S16. XRD patterns (reflection mode) of Mo_2TMB_2 (*TM* = Fe (*a*), Co(*b*) and Ni(*c*)) electrodes before (*black*) and after OER (*colored*). Calculated patterns of target and secondary phases also colored correspondingly.

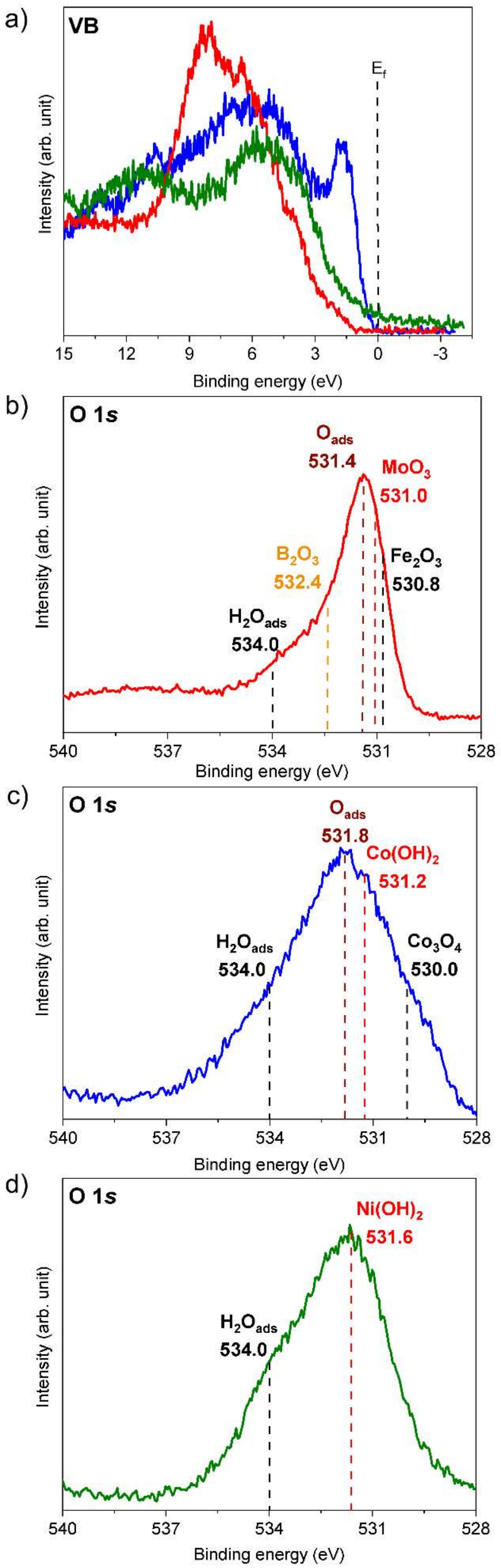

Figure S17. Valence band (*a*) and O 1*s* core level XP spectra of Mo_2FeB_2 (*red, b*), Mo_2CoB_2 (*blue, c*) and Mo_2NiB_2 (*green, d*) after OER (pre-treatment using protocol 1). The binding energies of adsorbed water (H_2O_{ads}), adsorbed O (O_{ads}), carbonate CO_3^{2-} as well as lattice oxygen in MoO_3, Fe_2O_3, Co_3O_4, B_2O_3, $Ni(OH)_2$ and $Co(OH)_2$ are taken from Refs. 1-5, 7-10.

Table S1. Optimized atomic coordinates for Mo_2TMB_2 (*TM* = Fe, Co, Ni).

**Element** (Wyckoff position)	**Atomic coordinates**		
	x/a	***y/b***	***z/c***
**Mo_2FeB_2** (*P*4/*mbm*, *a* = 5.7905(2) Å, *c* = 3.1462(2) Å)			
Mo (4*h*)	0.6778	0.1778	1/2
Fe (2*a*)	0	0	0
B (4*g*)	0.1129	0.6129	1/2
**Mo_2CoB_2** (*Immm*, *a* = 3.1658(2) Å, *b* = 4.5679(3) Å, *c* = 7.0907(4) Å)			
Mo (4*j*)	1/2	0	0.2996
Co (2*a*)	0	0	0
B (4*h*)	0	0.2066	1/2
**Mo_2NiB_2** (*Immm*, *a* = 3.1846(1) Å, *b* = 4.5603(2) Å, *c* = 7.0872(2) Å)			
Mo (4*j*)	1/2	0	0.3005
Ni (2*a*)	0	0	0
B (4*h*)	0	0.2037	1/2

References

[1] Moulder, J. F.; Stickle, W. F.; Sobol, P. E.; Bomben, K. D. *Handbook of X-ray Photoelectron Spectroscopy Studies*. Physical Electronics, Inc. Eden Prairie, Minnesota, USA, 1995.

[2] Coultas, S. J.; Counsell, J. D. P.; Gerrard, N. First Row Transition Metals Fe, Co, Ni, Cu, and Zn Analyzed by XPS Using Monochromatic Ag Lα X Rays. *Surf. Sci. Spectra* **2021**, 28 (2), No. 024004.

[3] McIntyre, N. S.; Johnston, D. D.; Coatsworth, L. L.; Davidson, R. D.; Brown, J. R. X-Ray Photoelectron Spectroscopic Studies of Thin Film Oxides of Cobalt and Molybdenum. *Surf. Interface Anal.* **1990**, 15 (4), 265-272.

[4] Joyner, D. J.; Johnson, O.; Hercules, D. M. A Study of the Iron Borides. 1. Electron Spectroscopy. *J. Am. Chem. Soc.* **1980**, 102 (6), 1910-1917.

[5] Armelao, L.; Barreca, D.; Gross, S. Sol-Gel and CVD Co_3O_4 Thin Films Characterized by XPS. *Surf. Sci. Spectra* **2021**, 8 (1), 14-23.

[6] Lyons, M. E. G.; Brandon, M.P. The Oxygen Evolution Reaction on Passive Oxide Covered Transition Metal Electrodes in Aqueous Alkaline Solution. Part I - Nickel. *Int. J. Electrochem. Sci.* **2008**, *3* (12), 1386-1424.

[7] Cole, K. M.; Kirk, D. W.; Thorpe, S. J. Co_3O_4 Nanoparticles Characterized by XPS and UPS. *Surf. Sci. Spectra* **2021**, 28 (1), No. 014001.

[8] Cole, K. M.; Kirk, D. W.; Thorpe, S. J. $Co(OH)_2$ Powder Characterized by X-Ray Photoelectron Spectroscopy (XPS) and Ultraviolet Photo-electron Spectroscopy (UPS*). Surf. Sci. Spectra* **2020**, 27 (2), No. 024013.

[9] Mansour, A. N. Characterization of β-$Ni(OH)_2$ by XPS. *Surf. Sci. Spectra* **1994**, 3 (3), 239-246.

[10] Mansour, A. N.; Melendres, C. A. Characterization of α-$Ni(OH)_2$ by XPS. *Surf. Sci. Spectra* **1994**, 3 (3), 255-262.
